# Evaluating the effects of synthetic POM cycles and NAD^+^ kinase expression on fatty alcohol production in *Saccharomyces cerevisiae*

**DOI:** 10.1371/journal.pone.0333299

**Published:** 2025-09-29

**Authors:** Bonnie A. McNeil, Charfeddine Khalifa, Anagha Krishnan, David T. Stuart

**Affiliations:** Department of Biochemistry, University of Alberta, Edmonton, Alberta, Canada; Chinese Academy of Sciences, CHINA

## Abstract

Efficient regeneration of NADPH can be a limiting factor for anabolic processes in engineered microbial cells. We tested the ability of four distinct Pyruvate-Oxaloacetate-Malate “POM” cycles composed of *Saccharomyces cerevisiae* pyruvate carboxylase (*PYC1* or *PYC2*), malate dehydrogenase (*‘MDH1* or *‘MDH2*), and malic enzyme (*sMAE1*) to improve NADPH regeneration. Only the *PYC1*, *‘MDH2*, *sMAE1* combination increased the titer of fatty alcohols produced by engineered *S. cerevisiae* indicating that not all combinations of POM cycle enzymes could drive this pathway. Metabolomic analysis revealed that introduction of the POM cycle altered the concentration of intermediates in amino acid biosynthetic pathways and the trichloroacetic acid cycle suggesting that the POM cycle had wider effects than previously anticipated. Overexpression of the endogenous NAD^+^ kinases *UTR1*, *YEF1*, and a cytosolic version of *POS5* were also tested. Only expression of *POS5c* resulted a significant increase in fatty alcohol titer. In these minimally engineered strains, combined overexpression of the *PYC1*, ‘*MDH2*, *sMAE1* POM cycle and *POS5c* did not further increase titers. These findings indicate that more extensive metabolomic and proteomic investigations are required to identify combinations of enzymes that will yield an optimal increase in NADPH to meet anabolic demands without imposing excessive metabolic burden or disrupting pathways that might compromise bioproduct synthesis.

## Introduction

Metabolic engineering of microbial strains can perturb metabolite abundance and redox-cofactor availability creating bottlenecks that hinder growth and negatively influence the titers, rates and yields (TRY) of desired products [1–3]. In particular, upregulation of biosynthetic processes can increase demand for the reduced form of Nicotinamide Adenine Dinucleotide Phosphate (NADPH), which plays a vital role in driving many anabolic reactions [4]. Therefore, achieving high titers of products through metabolic engineering relies on a steady supply of NADPH. This can be provided: (i) from NADP^+^ by NADP^+^-dependent enzymes (ii) from NAD^+^ or NADH kinases and (iii) by catalyzing the reaction NADH + NADP^+^ ⇄ NAD^+^ + NADPH through the activity of transhydrogenases [5].

An increased demand for NADPH is particularly salient in the production of fatty acid-based chemicals. Fatty acid synthesis requires 14 molecules of NADPH for the production of one C16 acyl chain. Additionally, the terminal enzymes employed in many conversion processes such as the production of fatty alcohols by fatty acyl-CoA reductases (FAR) also rely on NADPH as cofactors. Approaches to improving NADPH generation by microbial cells have clustered around three themes: (i) elimination or knockdown of competitive pathways, (ii) altering cofactor preference of overexpressed enzymes, (iii) and the transformation of NAD^+^ to its analog NADP^+^ [5]. The first two approaches can be uniquely targeted to specific production pathways. However, efficiently transforming NAD^+^ to NADP^+^, through the overexpression of transhydrogenases, transhydrogenase-like shunts or NAD^+^ kinases is a general mechanism to improve NADPH availability that can be applied to a wide variety of bioengineering projects without altering the net carbon flux.

In many organisms, NAD(P)^+^-dependent transhydrogenases play an important role in maintaining cellular redox poise and regenerating NAD^+^ and NADPH by catalyzing the reaction: NADH + NADP^+^ ⇄ NAD^+^ + NADPH [6]. Yeasts do not encode NAD(P)^+^-dependent transhydrogenases and rely on other routes to regenerate NADPH [7]. Heterologous expression of membrane-bound and soluble transhydrogenases from *Azotobacter vinelandii* and *Escherichia coli* in *Saccharomyces cerevisiae* have been unsuccessful in generating NADPH [8–10]. Therefore, expression of bacterial transhydrogenases is thought not to be a viable route for NADPH production in *S. cerevisiae*.

**Fig 1:**
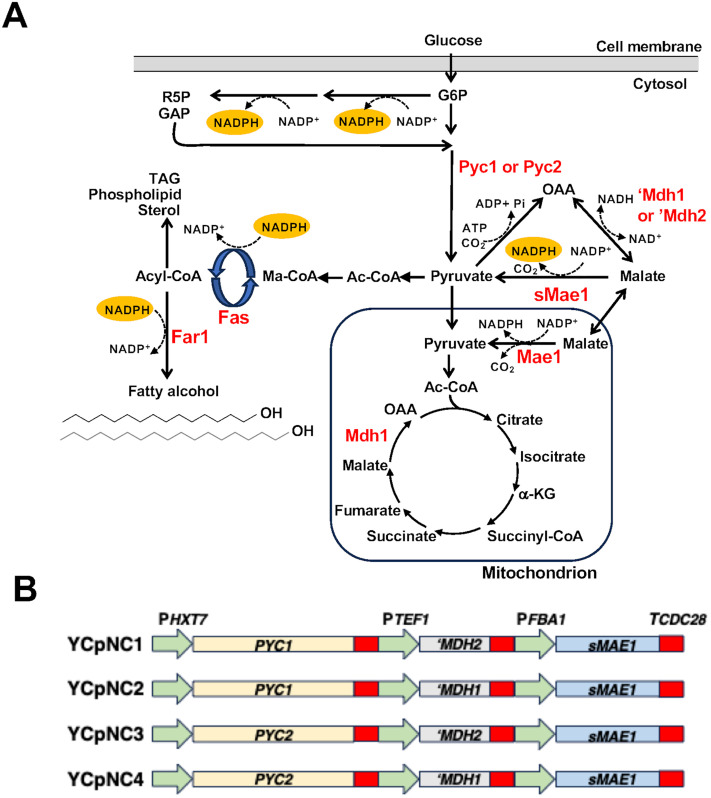
Synthetic POM cycles in the context of cellular metabolic pathways. **(A)** Schematic representation of enzymes and intermediates in the synthetic POM cycles and the major points where NADPH is produced or consumed in the production of fatty alcohols. **(B)** The composition of each of the plasmids containing synthetic POM cycles. All coding sequences are driven by heterologous promoters (indicated above the green arrows). The coding sequences for the *MDH1*, *MDH2* and *PYC1, PYC2* genes retain the native 3’UTR (red box). The sMAE1 coding sequence has a 3’UTR from *CDC28* (red). Each of the depicted gene arrays is carried on the centromeric plasmid YCplac111. Abbreviations: FAS, Fatty acyl-synthetase, FAR, Fatty acyl reductase, G6P Glucose-6-phosphate, R5P ribulose 5-phosphate, GAP glyceraldehyde 3-phosphate, OAA, oxaloacetate, Ac-CoA acetyl-CoA, Ma-CoA malonyl CoA, α-KG alpha ketoglutarate, TAG triacyclglycerols.

Some oleaginous yeasts encode a cytosolic malic enzyme and can utilize a three-enzyme transhydrogenase-like cycle referred to as the pyruvate-oxaloacetate-malate or “POM” cycle [11]. In the POM cycle, pyruvate is converted to oxaloacetate by pyruvate carboxylase (Pyc), oxaloacetate is then converted to malate, by malate dehydrogenase (Mdh), and malate is converted back to pyruvate by the malic enzyme (Mae) (Fig 1A). The net stoichiometry of these enzymatic reactions, ATP + NADH + NADP^+^ → +ADP + P_i _+ NAD^+^ + NADPH, is essentially identical to that of the bacterial transhydrogenase except that ATP is also utilized [6]. The presence of a cytosolic malic enzyme is important as NADPH cannot freely cross the mitochondrial membrane. Also salient is that the cycle regenerates pyruvate, a key intermediate in many cellular processes and does not alter the net carbon flux. The lack of a cytosolic malic enzyme in *S. cerevisiae* means that a fully cytosolic POM cycle does not naturally exist in these yeasts. Instead, much of the cytosolic reducing power is provided through the glucose-6-phosphate dehydrogenase (Zwf1) and 6-phosphogluconate dehydrogenase (Gnd1) steps of the pentose phosphate pathway, the cytosolic NADP^+^-specific isocitrate dehydrogenase (Idp2) and the NADP^+^-dependent aldehyde dehydrogenase (Ald6) [12]. Mitochondrial NADPH is generated through the mitochondrial NAD^+^ kinase (Pos5), through the activity of aldehyde dehydrogenases (Ald4/5) and through the activity of mitochondrial NADP^+^-dependant malic enzyme (Mae1) [13].

The overexpression of endogenous enzymes to create synthetic POM cycles has been employed in *S. cerevisiae* to counter redox imbalance imposed by the introduction of synthetic xylose metabolism, isobutanol production pathways and to increase the pools of NADPH in different cellular compartments [12,14–16]*.* These POM cycles have employed the endogenous protein isoforms Pyc2 and Mdh2 and either the endogenous malic enzyme (Mae1) or a truncated cytosolic version (sMae1). More recently, a POM cycle was established utilizing Pyc1, Mdh3 and a heterologous malic enzyme from *Rhodosporidium toruloides* (RtME) to reprogram *S. cerevisiae* from alcoholic fermentation to lipogenesis [17]. However, a comparison of the efficacy of the various shunts has not been reported.

We elected to generate and test POM cycles composed of endogenous isoforms of pyruvate carboxylase encoded by *PYC1* or *PYC2*, malate dehydrogenase encoded by *MDH1* or *MDH2* and the sole malic enzyme encoded by *MAE1*. Endogenous enzymes were selected based on the high likelihood that they would be successfully expressed in *S. cerevisiae*. *MAE1* was included in all combinations but was modified by removal of the mitochondrial signal sequence to ensure it would be localized to the cytosol [18]. *S. cerevisiae* encodes three isoforms of malate dehydrogenase each with distinct cellular functions. Mdh1 is a mitochondrial enzyme operating in the tricarboxylic acid (TCA) cycle and the malate/aspartate shuttle primarily to synthesize oxaloacetate from malate [19,20]. Mdh2 is cytosolic, functions primarily in gluconeogenesis to make malate from oxaloacetate, and is targeted for proteolysis in glucose containing media [21–24]. Mdh3 is peroxisomal and functions in the glyoxylate cycle [25]. *MDH1* and *MDH2* were included in our POM cycle combinations as they have been reported to have high catalytic activity and to accumulate in the cytosol [26]. *S. cerevisiae* also encodes two isoforms of pyruvate carboxylase encoded by *PYC1* and *PYC2*. The distinct roles of Pyc1 and Pyc2 in *S. cerevisiae* are less clear, however they are differentially regulated in response to carbon and nitrogen sources [27–29]. The effectiveness of different combinations of Mdh and Pyc isoforms when combined with Mae1 in the cytosol as part of synthetic POM cycles has not been reported.

Transformation of NAD^+^ to NADP^+^ by *S. cerevisiae*, is naturally accomplished through the activity of NAD^+^ kinases Yef1, Utr1 and Pos5 [30,31]. *POS5* encodes a mitochondrial kinase that preferentially phosphorylates NADH [13]. Both the endogenous Pos5 and a modified isoform lacking the mitochondrial signal sequence have been used successfully to counter redox imbalance in metabolic engineering studies [32–34]. *YEF1* and *UTR1* encode cytosolic paralogs that display activity towards both NAD^+^ and NADH. Overexpression of *UTR1* improved ethanol production in an engineered strain of *S. cerevisiae* [35], however, to the best of our knowledge overexpression of *YEF1* has not been investigated for its effects on biosynthetic processes and a general comparison of the effectiveness of overexpression of these NAD^+^ kinases on biosynthetic processes is lacking.

The aim of this study is to compare the efficacy of these general methods for using the more abundant (NAD^+^ and NADH) cofactors to generate NADPH for fatty acid-based chemical production in *S. cerevisiae*. We established and tested four synthetic POM cycles composed of distinct combinations of malic enzyme, pyruvate carboxylase and malate dehydrogenase isoforms for their effects on fatty alcohol production. In addition, we investigated the effects of cytosolic overexpression of the three endogenous NAD^+^ kinases on fatty alcohol production. Finally, we investigated whether the best performing POM cycle could have additive effects when combined with the overexpression of NAD^+^ kinases on fatty alcohol production in *S. cerevisiae.*

## Methods

### Strains, media and cultivations

**E. coli DH5*α*was used for the construction and propagation of all plasmid constructs. *E. coli* strains were cultured at 37°C in lysogeny broth (LB) supplemented with ampicillin (100 µg/mL). *S. cerevisiae* strains W303 *MAT*a ade2−201 his3−11,15 leu2−3,112 trp1−1 ura3−1 can1** and BY4741 *MATa his3Δ*1 leu2*Δ*0 met15*Δ*0 ura3*Δ*0** were cultured in either YEPD medium (Yeast extract 10 g/L, peptone 20 g/L, D-glucose 20 g/L) or synthetic defined media (SD) lacking leucine (-leu), uracil (-ura), or both leucine and uracil (-leu -ura) as appropriate (20 g/L glucose, 5 g/L ammonium sulfate, 1.7 g/L yeast nitrogenous base (YNB) without amino acids and 2 g/L of amino acid drop out mix). SD-KAc medium is SD medium with 2% potassium acetate replacing glucose. SD-Asp medium is SD with 0.5% aspartic acid replacing the ammonium sulfate.

### Plasmid and strain construction

Plasmids and strains used in this study are listed in Table 1; oligonucleotides utilized for plasmid and strain construction are listed in S1 Table. The *PYC1, PYC2, MDH1, and MDH2* open reading frames and transcriptional terminators, the open reading frame of *MAE1*, the *TEF1*, *HXT7* and *FBA1* promoters and *CDC28* 3’UTR were amplified from *S. cerevisiae* W303 genomic DNA using the appropriate oligonucleotide primers (S1 Table). The oligonucleotides were designed to delete amino-terminal coding sequence implicated in protein instability (*MDH2*) or targeting the enzyme to the mitochondria (*MDH1, MAE1*) and to introduce necessary homologies and restriction sites for assembling the genes into distinct combinations for POM cycle expression (Fig 1B). All gene fragments were assembled into the yeast/*E. coli* shuttle vector, YEplac181 [36] via Gibson isothermal assembly [37] thus creating four distinct POM cycle expressing cassettes (Table 1). The gene cassettes were then excised and transferred to the centromere containing plasmid YCplac111. Single gene control vectors were generated both with and without a carboxyl-terminal MYC epitope tag via Gibson assembly utilizing oligonucleotides listed in S1 Table. The YEplac195bb-P*PGK1*-ScFAR plasmids were constructed by inserting a codon optimized *Mus musculus FAR1* (Sc*FAR*) into YEplac195-P*PGK1* [38]. Genes encoding *S. cerevisiae* NAD^+^ kinases *UTR1*, *YEF1,* and *POS5c* under the regulation of a *PYK1* promoter were subsequently assembled into this vector to yield a vector over expressing each of the NAD^+^ kinases and ScFAR1.

**Table 1:** Plasmids and strains used in this study.

Plasmid	Characteristics	Reference
YEplac181	AmpR; High copy *LEU2 S. cerevisiae – E. coli* shuttle vector	[36]
YEplac181- PHXT7*- P*FBA1*-MAE1	AmpR; YEplac181 derivative containing the *S. cerevisiae HXT7* and *FBA1* promoters separated by AvrII and SmaI restriction sites and *sMAE1*	This work
YEpNC1	AmpR; YEplac181 derivative containing P*HXT7*-*PYC1* – P*TEF1-‘MDH2* – P*FBA1*-s*MAE1*	This work
YEpNC2	AmpR; YEplac181 derivative containing P*HXT7*-*PYC1* – P*TEF1-‘MDH1* – P*FBA1*-s*MAE1*	This work
YEpNC3	AmpR; YEplac181 derivative containing P*HXT7*-*PYC2* – P*TEF1-‘MDH2* – P*FBA1*-s*MAE1*	This work
YEpNC4	AmpR; YEplac181 derivative containing P*HXT7*-*PYC2* – P*TEF1-‘MDH1* – P*FBA1*-s*MAE1*	This work
YCplac111	AmpR; CEN plasmid LEU2 *S. cerevisiae* – *E. coli* shuttle vector	[36]
YCplac111-fMAE1-CDC28t	AmpR; YCplac111 derivative containing a fragment of *MAE1* and the *CDC28* terminator sequence	This work
YCpNC1	AmpR; YCplac111 derivative containing P*HXT7*-*PYC1* – P*TEF1-‘MDH2* – P*FBA1*-s*MAE1*	This work
YCpNC2	AmpR; YCplac111 derivative containing P*HXT7*-*PYC1* – P*TEF1-‘MDH1* – P*FBA1*-s*MAE1*	This work
YCpNC3	AmpR; YCplac111 derivative containing P*HXT7*-*PYC2* – P*TEF1-‘MDH2* – P*FBA1*-s*MAE1*	This work
YCpNC4	AmpR; YCplac111 derivative containing P*HXT7*-*PYC2* – P*TEF1-‘MDH1* – P*FBA1*-s*MAE1*	This work
YCpMAE1	AmpR; YCplac111 derivative containing P*FBA1*-s*MAE1*	This work
YCpPYC1	AmpR; YCplac111 derivative containing P*HXT7*-*PYC1*	This work
YCpPYC2	AmpR; YCplac111 derivative containing P*HXT7*-*PYC2*	This work
YCp’MDH1	AmpR; YCplac111 derivative containing P*TEF1-‘MDH1*	This work
YCp’MDH2	AmpR; YCplac111 derivative containing P*TEF1-‘MDH2*	This work
YCpPYC1-myc	AmpR; YCplac111 derivative containing P*HXT7*-*PYC1-myc*	This work
YCpPYC2-myc	AmpR; YCplac111 derivative P*HXT7*-*PYC2-myc*	This work
YCp’MDH1-myc	AmpR; YCplac111 derivative containing P*TEF1-‘MDH1-myc*	This work
YCp’MDH2-myc	AmpR; YCplac111 derivative containing P*TEF1-‘MDH2-myc*	This work
YEpScFAR	AmpR; YEplac195 derivative containing P*PGK1* driving expression of the *Mus musculus* FAR1 gene codon optimized for expression in *S. cerevisiae*	This work
YEpPOS5c/ScFAR	AmpR; YEplac195 derivative containing P*PYK1* driving expression of a cytosolic version of Pos5 and P*PGK1*-*ScFAR*	This work
YEpUTR1/ScFAR	AmpR; YEplac195 derivative containing P*PYK1* driving expression of *UTR1* and P*PGK1*-*ScFAR*	This work
YEpYEF1/ScFAR	AmpR; YEplac195 derivative containing P*PYK1* driving expression of *YEF1* and P*PGK1*-*ScFAR*	This work
pRS303-PGK1-FAS1	AmpR; pRS303 derivative containing P*PGK1* driving expression of *FAS1*	Courtesy of XiaoDong Liu
**Strains**	**Relevant Genotype**	**Source**
*S. cerevisiae* W303	*MATa leu2–3,112 trp1–1 can1–100 ura3–1 ade2–1 his3–11,15*	Open Biosystems
BMY01	W303 YCplac111	This work
BMY02	W303 YCpNC1	This work
BMY03	W303 YCpNC2	This work
BMY04	W303 YCpNC3	This work
BMY05	W303 YCpNC4	This work
BMY06	W303 YCpMAE1	This work
BMY07	W303 + YCp’MDH1-myc	This work
BMY08	W303 + YCp’MDH2-myc	This work
BMY09	W303 + YCpPYC1-myc	This work
BMY10	W303 + YCpPYC2-myc	This work
FAS1	W303 *fas1::PPGK1-FAS1-HIS3*	This work
BMY11	FAS1 YEpScFAR	This work
BMY12	FAS1 YCplac111 YEpScFAR	This work
BMY13	FAS1 YCpNC1 YEpScFAR	This work
BMY14	FAS1 YCpNC2 YEpScFAR	This work
BMY15	FAS1 YCpNC3 YEpScFAR	This work
BMY16	FAS1 YCpNC4 YEpScFAR	This work
BMY17	FAS1 YCpMAE1 YEpScFAR	This work
BMY18	FAS1 YCp’MDH1 YEpScFAR	This work
BMY19	FAS1 YCp’MDH2 YEpScFAR	This work
BMY20	FAS1 YCpPYC1 YEpScFAR	This work
BMY21	FAS1 YCpPYC2 YEpScFAR	This work
BMY27	FAS1 YCplac111 YEpUTR1/ScFAR	This work
BMY28	FAS1 YCplac111 YEpYEF1/ScFAR	This work
BMY29	FAS1 YCplac111 YEpPOS5c/ScFAR	This work
BMY30	FAS1 YCpNC1 YEpUTR1/ScFAR	This work
BMY31	FAS1 YCpNC1 YEpYEF1/ScFAR	This work
BMY32	FAS1 YCpNC1 YEpPOS5c/ScFAR	This work
DSY1718	*MATa his3Δ1 leu2Δ0 met15Δ0 ura3Δ0 mdh2::KanMX4*	Open Biosystems
DSY1724	*MATa his3Δ1 leu2Δ0 met15Δ0 ura3Δ0 pyc1::KanMX4 pyc2::NatMX6*	This work

For overexpression of *FAS1* a 3430 bp DNA fragment encompassing the 5’ coding sequence of *FAS1* was ligated behind a *PGK1* promoter fragment (P*PGK1*) in integrating vector pRS303. The vector was digested with PacI to target integration to *FAS1* thus placing the chromosomal *FAS1* coding sequence under control of the *PGK1* promoter. The *PYC2* coding sequence in DSY 1724 was replaced with a NatMX6 cassette using PCR mediated homologous recombination [39] with oligonucleotides PYC2D5 and PYC2D3 (S1 Table). All plasmids were verified by sequencing. Constructs were introduced into *S. cerevisiae* by lithium acetate mediated transformation [40] selecting for transformants on the appropriate synthetic medium. All resulting strains were verified by PCR amplification of diagnostic fragments from total chromosomal DNA.

### Protein extraction and western blotting

Control strain BMY01 and strains BMY07-BMY10 expressing MYC tagged proteins were cultured overnight in SD -leu medium, diluted 1:25 in fresh YEPD and incubated at 30°C until an OD_600_ of 0.5 was achieved. Total protein was extracted from 10 mL of culture via a trichloroacetic acid extraction [41]. Protein concentrations were determined by BCA assay [42]. Protein samples (20 µg) were separated by electrophoresis using 8% SDS-polyacrylamide gels and transferred to a polyvinylidene difluoride membrane (Immobilon-P, Millipore). The membrane was probed with anti-MYC mouse monoclonal antibody 9E10 (MMS-150R, Covance Laboratories) diluted 1:10,000 in TBST (3.0 g/L Tris-base, 8.0 g/L NaCl, 0.2 g/L KCl, pH 7.5, 0.2% Tween 20) supplemented with 5% skim milk powder, followed by washing with TBST and incubation with HRP-conjugated goat anti-mouse IgG (1706516 Biorad Labs) diluted 1:5000 prior to visualization with enhanced chemiluminescence. The membrane was stripped and re-probed with mouse monoclonal anti-PSTAIRE (P7962, Sigma-Aldrich) also diluted 1:10,000 in TBST + 5% skim milk powder to detect Cdc28 as a loading control. Primary antibody was detected with HRP-conjugated goat anti-mouse IgG (1706516 Biorad Labs) diluted 1:5000 prior to visualization with enhanced chemiluminescence.

### RNA extraction and RT-qPCR analysis

RNA was prepared as described [43]. 5 µL of each RNA sample was separated on a 1% bleach gel [44] for assessment of RNA quality. Purity and concentration of RNA samples were determined spectrophotometrically. Nucleic acid preparations were digested with RNase-free DNase I and cDNA synthesis was performed using 1 µg of total RNA and iScript reverse transcriptase supermix according to manufacturer’s protocol. No-RT controls were performed with the omission of the RT. qPCR reactions were in a total volume of 10 µL containing 10 pmol of forward and reverse primers (listed in S1 Table), 5.0 µL 2X iQ SYBR Green Supermix (Bio-Rad) and 1.25 µL of the RT reaction as template. A three-cycle amplification was performed (95°C for 10 s, 54°C for 15 s, 72°C for 20 s) with melt curve (ramping from 72°C to 95°C) using the Rotor-Gene Q, real-time PCR cycler (Qiagen). No-RT and no-template controls were included and all unknowns were tested in technical triplicates. Actin-specific primers were used to normalize the data. Amplification efficiency of all primer pairs was tested prior to use in RT-qPCR and were found to be between 90–105% efficiency.

### Fatty alcohol extraction and analysis

Cell cultures (25 mL) were overlaid with dodecane (2.5 mL) spiked with a pentadecanol internal standard. Following the 72-hour growth period, the culture was pelleted by centrifugation (2800 x g, 5 min, 4^o^C). The dodecane layer was isolated and then either used directly or 100 µL of the upper dodecane phase was diluted in 900 µL ethyl acetate. A 1 μL aliquot was analyzed via GC-FID using an Agilent 6890 series GC System equipped with a CP-Wax 58 FFAP CB column (25m x 320 μm x 0.2 μm, Agilent, USA). Helium was used as a carrier gas, with a constant flow of 1 mL/min. The temperature of the inlet was 240°C. The following program was applied: 50°C for 1 minute, increase 25°C/minute to 170°C followed by 10°C/min to 240°C, hold at 240°C for 7 minutes. The internal standards were used to determine product yields, in conjunction with calibration curves and known relative response factors. Mean values were determined from three independent biological replicates unless otherwise stated.

### NADPH/NADP^+^ analysis

*S. cerevisiae* cultures harboring an empty vector or POM cycle encoding plasmid were adjusted to 1 x 10^7^ cells/mL, cooled on ice, collected by centrifugation (3000 x g, 5 min), washed in ice cold water, resuspended in 500 µL extraction buffer and stored at -80^o^C overnight. Subsequently, cell pellets were thawed on ice, NADP^+^ and NADPH concentrations were determined by colorimetric assay (MAK479, Millipore-Sigma, Canada) following the manufacturer directions. Statistical analysis was performed by application of a 1-way ANOVA test and Tukey HSD was applied as a post-hoc test using Prism graphpad (Dotmatics).

### Metabolomic analysis

Triplicate samples of *S. cerevisiae* cultured in SD -leu medium (15 mL) at 30^o^C with agitation at 200 rpm, harboring either the empty vector plasmid YCplac111 or YCpNC2 at OD_600_ = 0.8 were cooled on ice and harvested by centrifugation for 3 min at 3000 x g. The cell pellets were washed in ice cold water, frozen in liquid nitrogen and stored at -80^o^C prior to analysis. 200 µL of 1:1 (v/v) methanol: water was added into each of the individual sample tubes. The samples were lysed by 5 cycles of vortexing with glass beads for 1 min, and cooling on ice between bursts of vortexing. 800 µL of 1:1 (v/v) methanol: water was added into each of the tubes. The tubes were vortexed for another 10 min and centrifuged at 15,000 x g for 10 min at 4°C. All the supernatants were transferred into new vials and dried down. The samples were re-dissolved in 41 µL of water. The total concentrations of samples were determined by NovaMT Sample Normalization kit. All the samples were diluted to 2 mM before labeling. The samples were split into four aliquots for different labeling methods, backup and preparation of pooled sample. The aliquot for preparing pooled sample from each individual sample was combined and mixed thoroughly to generate the pooled sample, which was used as the reference.

For each aliquot of sample for amine-/phenol- labeling LC-MS grade water was added to dilute the sample and 25 µL of sample was used for labeling. The dansyl (DnsCl) and dimethylaminophenacyl (DmPA), labeling protocol was performed largely as described [45,46]. Briefly, 12.5 µL of 0.5 M NaHCO_3_/Na_2_CO_3_ buffer and 37.5 µL of ^12^C2-DnsCl (for the individual samples and the pooled sample) or ^13^C2-DnsCl (for the pooled sample) was added into samples. The samples were then vortexed and collected by centrifugation. The mixtures were incubated at 40°C for 45 minutes. labelling reactions were quenched by addition of 7.5 µL of 250 mM NaOH. The mixtures were incubated at 40°C for another 10 minutes. Finally, 30 µL of 425 mM formic acid was added.

The aliquot of sample for carboxyl labeling was dried and re-dissolved in 25 µL of 3:1 (v/v) acetonitrile: water. Briefly, 10 µL triethanolamine and 25 µL of ^12^C2-DmPA (for the individual samples and the pooled sample) or ^13^C2-DmPA (for the pooled sample) was added into samples. The mixtures were incubated at 80°C for 60 minutes. Reactions were quenched by addition of 40 µL of triphenylacetic acid. The mixtures were incubated at 80°C for another 30 minutes and the chemical isotope labeling procedure was complete. The ^12^C2-labeled individual sample was mixed with ^13^C2-labeled reference sample in equal amount. Prior to LC-MS analysis of the entire sample set, quality control (QC) sample was prepared by equal volume mix of a ^12^C-labeled and a ^13^C-labeled pooled sample. Metabolite analysis was performed using LC-MS with an Agilent 1290 HPLC instrument linked to Bruker Impact II QTOF Mass Spectrometer. The HPLC column was an Agilent Eclipse plus revered-phase C18 column (150 x 2.1 mm, 1.8 µm particle size). Separation was performed with buffers MPA 0.1% (v/v) formic acid in water, MPB 0.1% (v/v) formic acid in acetonitrile using a gradient t = 0 min, 25% B; t = 10 min, 99% B; t = 15 min, 99% B; t = 15.1 min, 25% B; t = 18 min, 25% B, Flow rate 400 µL/min, column Oven temp 40^o^C, MS data in the Mass range m/z 220–1000 were collected with an acquisition rate 1 Hz.

A total of 18 LC-MS data from 2-channel analysis (9 LC-MS data, including 3 QC in each channel) were first exported to.csv file with Bruker DataAnalysis 4.4. The exported data were uploaded to IsoMS Pro 1.2.15 for Data Quality Check and Data Processing. Statistical analysis and volcano plots were performed using MetaboAnalyst 6.0 (www.metaboanalyst.ca). Statistical analysis was performed on fatty alcohol and NADPH/NADP^+^ data using Prism graphpad (Dotmatics).

## Results

### Construction of synthetic POM cycles

The proposed mechanism of carbon shuttling and generation of NADPH by a POM cycle is shown in Fig 1A. Key to effectively engaging this cycle is over production of enzymes that have pyruvate carboxylase activity (Pyc), NADH dependent malate dehydrogenase activity (Mdh) and NADP^+^ dependent malate decarboxylase activity (Mae). The enzymes must be compartmentalized in the cytosol to have ready access to the same pool of intermediates and be able to regenerate NADPH making it available for cytosolic anabolic processes (Fig 1A).

Gene cassettes encoding four distinct sets of POM cycle enzymes were constructed in the Yeast/*E.coli* shuttle vector YCplac111 (YCpNC1–4; Table 1). Each cassette contains a truncated version of the *S. cerevisiae* malic enzyme gene *(sMAE1),* lacking the first 90 nucleotides encoding amino acids that form a mitochondrial targeting signal [18]. This variant of Mae1 localizes to the cytosol [18]. The truncated *MAE1* coding sequence was placed under the regulation of an *FBA1* promoter and a 3’ UTR sequence derived from *CDC28*. The cassettes also contain the gene sequences for either of the *S. cerevisiae* isoforms of pyruvate carboxylase (*PYC1* or *PYC2*) and an isoform of malate dehydrogenase (‘*MDH1* or ‘*MDH2*) resulting in four combinatorial arrangements (Fig 1B).

Differing from most other eukaryotic cells, *S. cerevisiae* encodes two isoenzymes of pyruvate carboxylase that are localized to the cytosol rather than a single mitochondrial enzyme. Expression of the isoforms is differentially regulated at the level of transcription depending on factors such as growth phase, carbon source and nitrogen source [27–29]. The *PYC1* and *PYC2* coding sequences were placed under the regulation of an *HXT7* promoter to limit differential transcriptional regulation but retained their native 3’ UTR regions (Fig 1B). Each protein isoform displays different allosteric properties with respect to acetyl-CoA activation and aspartate inhibition with Pyc1 displaying a higher degree of cooperativity [47]. In addition, Pyc2 reportedly displays lower abundance and reduced activity in glucose [27]. Therefore, it is reasonable to predict that despite providing the same promoter for *PYC1* and *PYC2* that the encoded isoforms may display differences in activity when incorporated into the synthetic POM cycles.

For the expression of malate dehydrogenase, we aimed to utilize versions that would be both cytosolic and stable in medium containing glucose as the carbon source. To achieve this, a nucleotide sequence encoding the 17 amino acid mitochondrial signal sequence was deleted from *MDH1* [20]. Mdh2 is subject to both transcriptional repression and degradation in the presence of glucose [22]; therefore, to stabilize Mdh2, the nucleotide sequence encoding 12 amino acids at the amino-terminus was deleted in the constructs used in this study. The coding sequences for both Mdh1 and Mdh2 were placed under the regulation of an *TEF1* promoter and both retained their native 3’ UTR sequences (Fig 1A). Each of the combinations of genes were assembled and inserted into a centromeric vector YCplac111 to form YCpNC1 (*PYC1, ‘MDH2 sMAE1*), YCpNC2 (*PYC1 MDH1 sMAE1*), YCpNC3 (*PYC2 MDH2 sMAE1*), YCpNC4 (*PYC2 MDH1 sMAE1)* (Fig 1B).

### Verification of protein isoform expression

**Fig 2:**
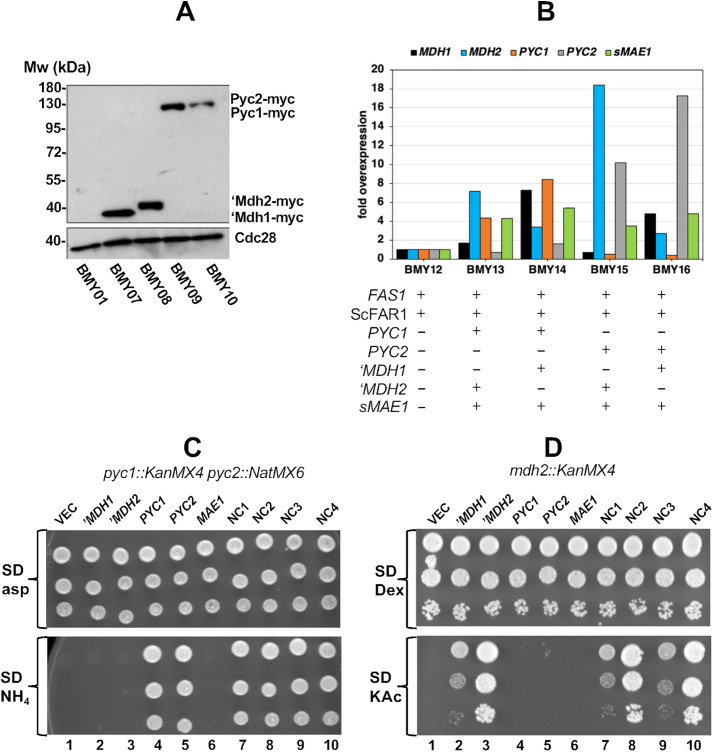
‘Mdh1, ‘Mdh2, Pyc1, Pyc2 and *MAE1* are expressed. **(A)** Western blot to detect ‘Mdh1, ‘Mdh2, Pyc1, Pyc2 expression using the anti-MYC antibody (upper panel). Cdc28 detected with anti-PSTAIRE antibody (lower panel) as a loading control. Molecular weights based on the migration of a pre-stained standard are indicated on the left side of the image. **(B)** Relative mRNA abundance corresponding to *MDH1, MDH2, PYC1, PYC2 and MAE1* from the POM cycle expressing strains determined by RT-qPCR. Heterologous gene additions to each strain are indicated. **(C)** Growth of *pyc1*::KanMX4 *pyc2*::NatMX6 strains on synthetic medium lacking leucine supplemented with 0.5% aspartic acid (upper panel), or 0.5% ammonium sulfate as a nitrogen source (lower panel). Each strain carried the indicated plasmid. Images were taken three days after spotting the cells to the plates. **(D)** Growth of *mdh2*::KanMX4 strains on synthetic medium supplemented with 2% glucose as a carbon source (Dex, upper panel) or 2% potassium acetate as a carbon source (KAc, lower panel). Each strain carried the indicated plasmid. Images were taken three days after spotting the cells to the indicated plates.

To confirm expression of the individual variants of pyruvate carboxylase and malate dehydrogenase, *S. cerevisiae* strains harboring centromeric vectors encoding the individual *PYC1*, *PYC2*, ‘*MDH1* and ‘*MDH2* variants that had been modified by the addition of a carboxyl-terminal MYC epitope were assayed by western blot. A control strain, BMY01 harboring an empty vector displayed no signal consistent with anti-MYC reactive proteins but the loading control Cdc28 can clearly be detected (Fig 2A, lane 1). In contrast, sample lanes loaded with extract from BMY07 and BMY08 displayed specific bands reacting with the anti-MYC antibodies migrating at 35 kDa and 40 kDa consistent with the predicted molecular weight of Mdh1 and Mdh2 (Fig 2A, lanes 2, 3). Extract from strains BMY09 and BMY10 displayed bands migrating at 130 kDa consistent with the predicted molecular weights of Pyc1 and Pyc2 (Fig 2A lanes 2, 3). Pyc1-MYC repeatably accumulated to a greater abundance than Pyc2. These results suggest that either Pyc1 is translated more efficiently or is more stable than Pyc2 under the culture conditions consistent with the previously observed higher abundance and activity of Pyc2 when cells are cultured in glucose supplemented medium [27]. The ‘Mdh1-MYC and ‘Mdh2-MYC variants tested displayed similar abundance to one another.

Having confirmed that the individual components of the synthetic POM cycles were being expressed we introduced plasmids encoding the POM cycle variations into *S. cerevisiae* XDY1. The open reading frames of *PYC1* and *PYC2* were both under the regulation of an *HXT7* promoter, which was expected to limit the differences in transcriptional regulation between the two but qPCR analysis demonstrated that the mRNA for *PYC2* (grey bars) accumulated to higher levels than the mRNA for *PYC1* (brown bars) (Fig 2B compare BMY13 - BMY15, BMY14 – BMY 16).

Interestingly, we observed that strains overexpressing *‘MDH1* (BMY14, BMY16) displayed a concurrent 3–4-fold increase in mRNA from the endogenous *MDH2* while this did not appear to be true for cells overexpressing *‘MDH2* (BMY13, BMY15) (Fig 2B). There is no published report of Mdh1 influencing the expression of *MDH2* but it is unlikely that this would be detrimental to the functioning of the synthetic POM cycle. The *sMAE1* mRNA (green bars) was constant among the four POM cycles.

The function of the *PYC1* and *PYC2* genes encoded on the POM cycle plasmids was tested by complementation of a *pyc1::KanMX4 pyc2::NatMX6* strain. The double mutant displays auxotrophy for aspartic acid and is viable and competent for growth on SD – leu medium supplemented with 0.5% aspartate as a nitrogen source independent of whether it carries an empty vector (Fig 2C, upper section, lane 1), or any of the plasmids encoding a POM cycle or individual enzymes of the POM cycle (Fig 2C upper section, lanes 2–10). In contrast, when aspartate is replaced with ammonium sulphate as the nitrogen source, cells carrying empty vector or any plasmid not encoding *PYC1* or *PYC2* failed to form colonies (Fig 2A, lower panel, lanes 1–3, 6). Cells that carried plasmids YCNC1 - YCNC4, encoding either *PYC1* or *PYC2* all displayed the ability to complement the *pyc1 pyc2* mutant consistent with the *PYC* gene on each POM cycle plasmid being functional.

The function of the *‘MDH1* and *‘MDH2* genes encoded by the POM cycle plasmids were tested by complementation of an *mdh2::KanMX4* strains growth defect on medium supplemented with acetate as the carbon source. The *mdh2::KanMX4* strain is viable and able to form colonies on SD – leu medium that has glucose as a carbon source independent of whether the strain carries an empty vector or a plasmid encoding the POM cycle genes individually or in combination (Fig 2D, upper panel lanes 1–10). In contrast, when glucose is replaced with 2% potassium acetate (KAc) as a carbon source only cells carrying a plasmid encoding *‘MDH2* or *‘MDH1* displayed colony formation (Fig 2D, lower panel, lanes). Complementation of *mdh2::KanMX4* growth on SD-KAc medium by *‘MDH1* yielded slow growth as has been previously reported [26].

### Effect of synthetic POM cycles on fatty alcohol production

As NADPH availability is extremely salient for the production of oleochemicals in microbial systems, we investigated the effects of the four synthetic POM cycles in an *S. cerevisiae* strain minimally engineered to produce fatty alcohol. These strains (BMY12 - BMY16; Table 1) overexpress Fatty acyl synthetase subunit *FAS1* under regulation of the strong constitutive *PGK1* promoter and express a codon-optimized version of the *Mus musculus* fatty acyl-CoA reductase (ScFAR). Increased expression of *FAS1* results in a coinciding increase in the levels of *FAS2* yielding an overall increase in the expression of the FAS complex responsible for fatty acyl-CoA synthesis [48]. This increases fatty acid biosynthesis activity, ultimately creating an increased demand for NADPH in the cells. As the reactions catalyzed by both the FAS complex and ScFAR1 enzyme are NADPH-dependent (Fig 1) we hypothesized the availability of NADPH may become limiting in this synthetic pathway and that the synthetic POM cycles would increase availability of NADPH and drive increased flux through the pathway resulting in an increased titer of fatty alcohols.

**Fig 3:**
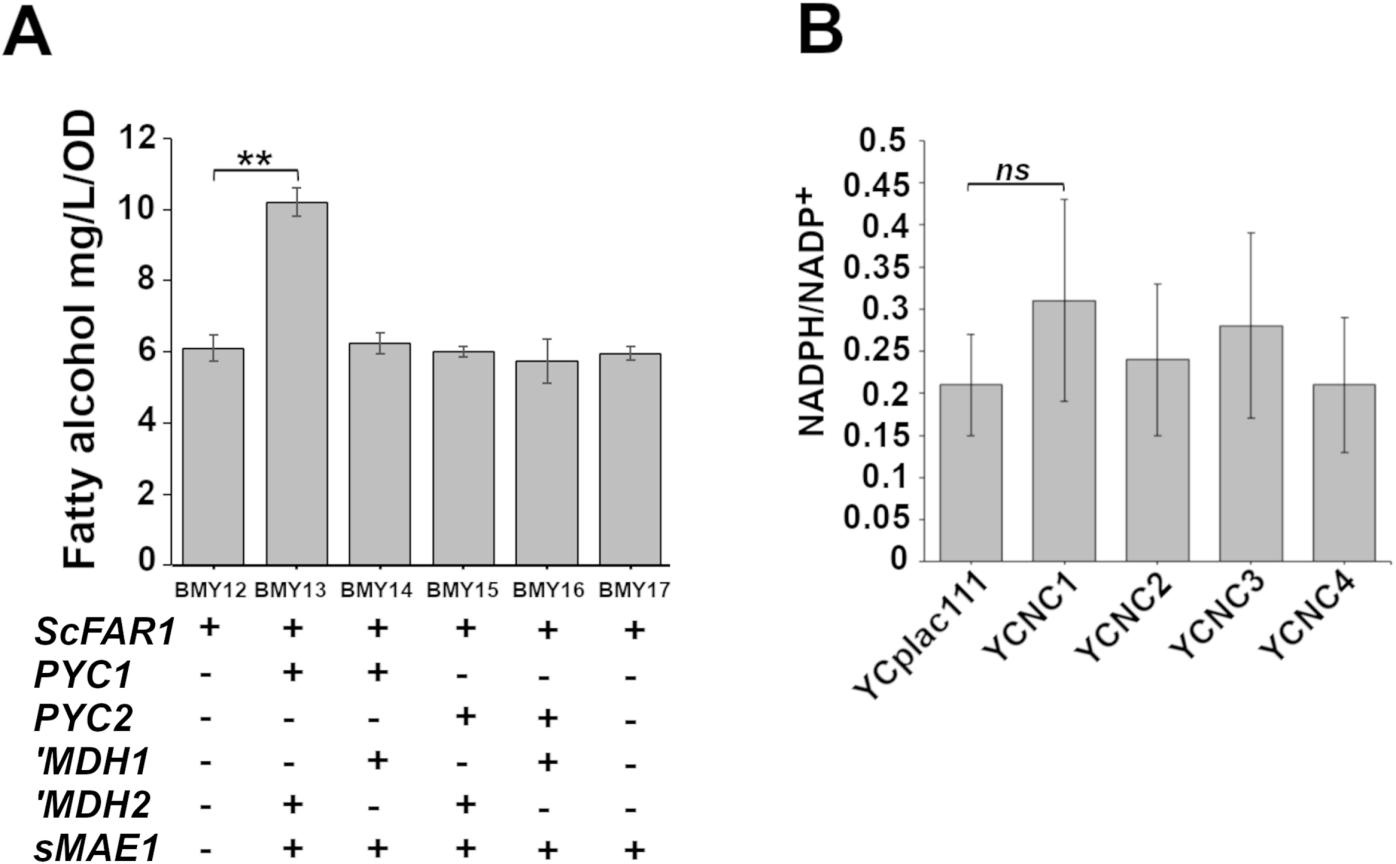
Effects of synthetic POM cycles on fatty alcohol production. **(A)** Fatty alcohol (combined hexadecanol and octadecanol) produced by the indicated strains. The shaded bars indicate mean fatty alcohol titers (mg/L) normalized to cell density (OD_600_) for samples collected after 72 hours of shake flask culture. Error bars indicate standard deviation from 3 independent biological replicates. **(B)** The ratio of NADPH/NADP^+^ from triplicate cultures of the indicated strain harboring an empty vector YCplac111, or the same vector encoding the indicated POM cycle enzyme cassettes. Data shown are mean values from 3 biological replicates each assayed with technical triplicates. Indicators of statistical significance: ns *p* > 0.05, * *p* ≤ 0.05, ** *p* ≤ 0.01, *** *p* ≤ 0.001.

Seven independent colonies from each transformation (BMY12 – BMY17) were tested for fatty alcohol production in shake flask culture. There was a high degree of variability in the titer of fatty alcohols produced among individual transformants carrying the same plasmid (Figure A and B in S1 File). The production characteristic of a transformant was clonal. When a high producing colony was streaked to isolate independent colonies all of those plasmid-bearing colonies consistently maintain high levels of production. Three single colonies were isolated from each of the highest producing candidates from the strains (BMY12 - BMY17) and tested for fatty alcohol production (Fig 3A). The BMY13 strain (*PYC1, ‘MDH2, sMAE1*) significantly increased fatty alcohol production by 40% over the control strain BMY12 (10.2 ± 0.5 mg/L/OD vs 6.1 ± 0.4 mg/L/OD, **p* *= 0.002) (Fig 3A). Interestingly, an ~ 20% decrease in fatty alcohol production was observed for the *PYC2* overexpressing strains BMY15 and BMY16 (Fig C in S1 File). However, when normalized for biomass density BMY15 (*PYC2, ‘MDH2, sMAE1*) and BMY16 (*PYC2, ‘MDH1, sMAE1*) produced fatty alcohol similar to the vector control strain BMY12, 6.1 ± 0.4 mg/L/OD (Fig 3A). Overexpression of the individual genes *sMAE1*, *‘MDH1, ‘MDH2, PYC1, PYC2* showed no increase in fatty alcohol over the control strain. (Figure S3 File).

### POM cycle expression has limited effects on steady state NADPH/NADP^+^ ratios

The engineered POM cycles were designed with the expectation that they could increase the regeneration of NADPH making more available for biosynthetic processes. To further investigate the metabolic effect of POM cycle expression an enzymatic assay was performed on whole cell extracts to determine the concentrations of NADP^+^ and NADPH. A comparison of the relative NADPH/NADP^+^ assayed in extracts prepared from the strains harboring an empty vector or each of the independent POM cycle plasmids indicated that no statistically significant increase in NADPH/NADP^+^ could be detected, NC1 *p* = 0.378, NC2 *p* = 0.981, NC3 *p* = 0.703, NC4 *p* = 1.000. It is likely that the considerable variation among the replicates (n = 9) contributed to this finding (Fig 3B). Measurable changes in NADPH in the system may be challenging to assay biochemically as elevating the NADPH is expected to promote NADPH driven anabolic pathways and the cells will seek homeostasis in the abundance of the redox cofactors.

### POM cycle influences amino acid biosynthetic pathways

The synthetic POM cycles are envisioned to take advantage of the relatively abundant NADH and ATP available in cells actively proliferating under aerobic fermentative conditions and generate elevated NADPH that could be used to drive anabolic pathways. While the enzymatic reactions can function cyclically starting with and regenerating pyruvate they do not act in isolation and the metabolites: pyruvate, malate and oxaloacetate are important intermediates in other pathways that could potentially be disrupted by their sequestration within a POM cycle. To determine whether the POM cycles tested in this study influenced the abundance of other intermediates in central metabolism we performed a limited metabolomic analysis on independent biological replicates (n = 3) that carried either the empty vector or the POM cycle cassette, NC1, that yielded the most significant increase in fatty alcohol production in our test strain.

**Fig 4:**
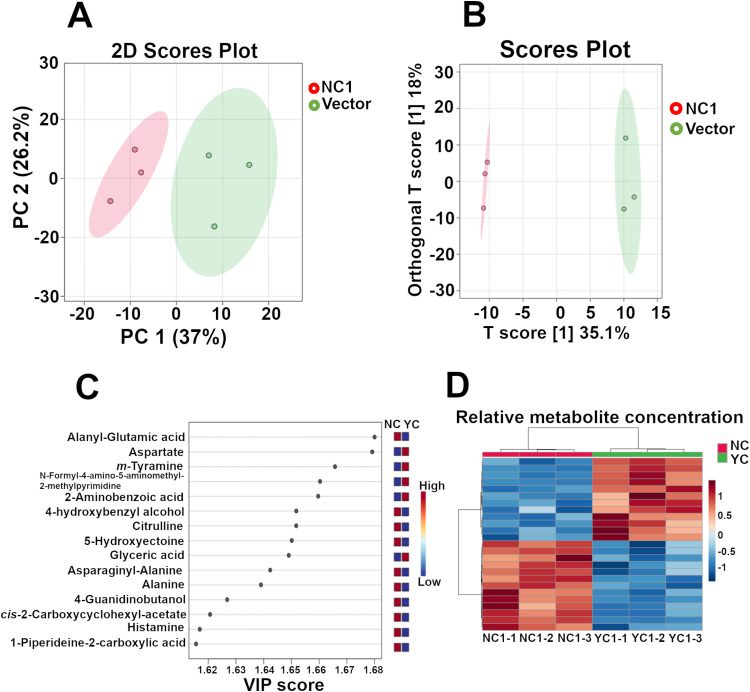
Effect of the POM cycles on selected metabolites. **(A)** Score plot of principal component analysis of intracellular metabolites extracted from triplicate cultures carrying either the empty vector YCplac111 (green) or the YCplac111 vector encoding the NC1 POM cycle cassette enzymes (red). **(B)** Orthogonal partial least squares discriminant analysis (OPLS-DA) scores plot of intracellular metabolites extracted from triplicate cultures carrying either the empty vector YCplac111 (green) or the YCplac111 vector encoding the NC1 POM cycle cassette enzymes (red). **(C)** Variable importance for the projection (VIP) scores of the top 25 metabolites with VIP scores >1. Metabolite name is on the left of the figure. The heat map indicator on the right of the figure indicates whether the indicated metabolite is elevated (red) or decreased (blue) in the empty vector strain (YC) or NC1 POM cycle expressing strain (NC). **(D)** Heatmap of metabolite abundance derived from a hierarchical clustering analysis of metabolites identified with high confidence in three biological replicate cultures of strains carrying the empty vector YCplac111 (green) or the NC1 POM cycle cassette (red).

Metabolite analysis identified 512 metabolites with high confidence that were used for further pathway analysis (S2 Table). Multi-variate analysis using unsupervised principal components analysis (PCA) indicated that significant differences were displayed between the NC1 expressing cultures and those with the empty vector control (Fig 4A). PC1 and PC2 give an explanation rate of 63.2% with PC1 accounting for 37% of the variation. Supervised orthogonal projections to latent structures discriminant analysis (OPLS-DA) was used to distinguish differentially abundant metabolites between the two groups. The OPLS-DA score graph demonstrated clear differences between the NC1 POM cycle expressing culture samples (red) and the cultures containing an empty vector only (green) based on the PC1 score 35% (Fig 4B). Variable importance for the projection (VIP) scores were plotted to gain insight into metabolite features contributing to separation between the POM cycle and control groups. This analysis yielded scores > 1 for amino acids and intermediates in the synthesis and degradation pathways for aspartic acid, alanine, arginine, and lysine (Fig 4C). The data were further summarized and dynamic changes investigated with hierarchical cluster analysis (HCA). The three independent replicates of the NC1 POM cycle producing strain (red) and the empty vector containing strain (green) were clustered together indicating relative homogeneity and reproducibility in the change among the top 50 metabolites in the analysis (Fig 4D).

**Fig 5:**
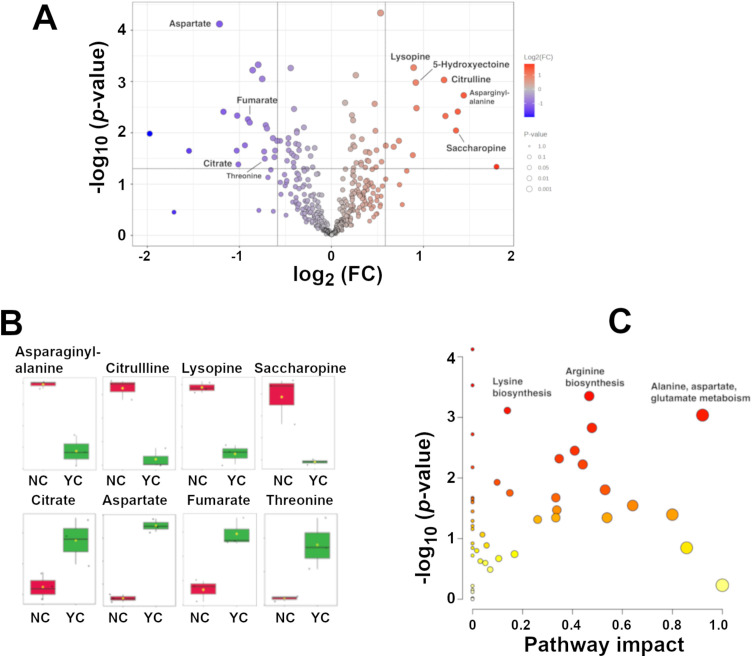
Amino acid metabolic pathways are influenced by the NC1 POM cycle over expression. **(A)** Volcano plot of metabolites identified with high confidence from strains carrying an empty vector or the NC1 POM cycle cassette. Metabolites displaying an increase (FC > 1.5, **p* *< 0.05) in the NC1 strain are displayed in red. Metabolites with reduced abundance in the NC1 strain are displayed in blue. Dotted lines indicate the FC and *p* value thresholds. **(B)**. Box and whisker plots of selected metabolites that displayed significant increase or decrease in the NC1 POM cycle producing strain (NC), red boxes, or empty vector producing strain (YC) green boxes. Individual data points are indicated by black dots, sample means are indicated by white diamonds within each box. **(C)** Pathway impact plot of metabolites identified displaying differential abundance in the NC1 POM cycle expressing strain relative to the empty vector. The size of each circle reflects the number of “hits” relevant to the pathway with red having higher and yellow having lower significance based on *p*-value score.

Differential analysis was employed to further examine differences among identified metabolites that display statistically significant changes in abundance between strains. As indicated by the volcano plot there were significant differences displayed among a subset of the positively identified metabolites. When using a fold change threshold FC > 1.5, **p* *< 0.05 a significant increase in abundance was displayed by 13 metabolites in the NC1 POM cycle expressing strains (Fig 5A, red scatter plot points). There were 21 metabolites displaying a significant decrease in the NC1 POM cycle expressing strain (Fig 5A, blue scatter plot points). Although OAA was not unambiguously detected, pyruvate and malate were clearly identified in the analysis and no significant difference between the strains in the concentration of either metabolite was detected (S2 Table). An increase in arginine biosynthesis intermediate citrulline (fold change 2.33 *p* = 0.00094) was detected along with, lysine biosynthetic pathway intermediates saccharopine (fold change 2.55 *p* = 0.040), and lysopine (fold change 1.85 *p* = 0.0014). Additionally, an increase in 5-hydroxyectoine (fold change 1.88 *p* = 0.007) which could be derived from aspartic acid catabolism was also detected (Fig 5A). Conversely, significant decreases in the abundance of aspartic acid (fold change 0.43 *p* = 0.00038), fumarate (fold change 0.53 *p* = 0.006) and citrate (fold change 0.49 *p* = 0.04) were detected (Fig 5, S2 Table). Hence, elevating the concentration of Pyc1 and Mdh2 and introducing malic enzyme to the cytosol may have unplanned consequences to off target metabolic pathways.

The metabolite set identified as high-confidence results were subsequently subjected to metabolic pathway analysis using Global Test as enrichment analysis and Relative-betweenness Centrality as topology analysis in MetaboAnalyst. The data, graphed as scatter-plot of the metabolites *p*-value against pathway impact, suggests perturbation of several metabolic pathways, most notably amino acid synthesis and metabolism pathways (Fig 5C). Other pathways including pyruvate and glutathione metabolism are summarized in S3 Table.

### Effect of NAD^+^ kinase expression on fatty alcohol production

**Fig 6:**
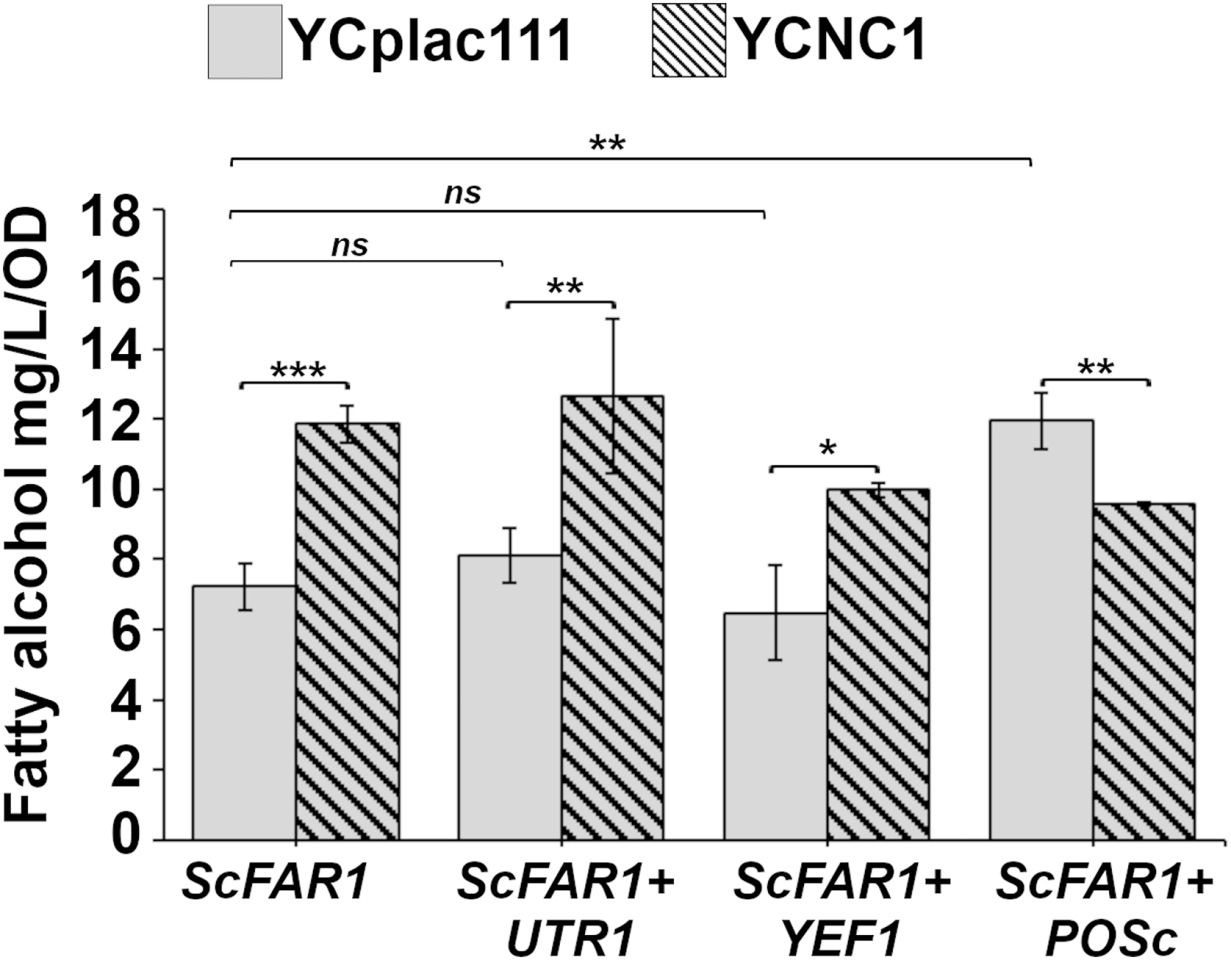
Effect of the NAD^+^ Kinases Utr1, Yef1, and Pos5c on fatty alcohol production. The X-axis label indicates the over produced NAD^+^ kinase. Fatty alcohol titers (mg/L/OD) for high producers of strains expressing NAD^+^ kinase without co-expression of the POM cycle encoded by YCpNC1 (grey bars) or with the expression of the NC1 POM cycle enzymes (hatched bars). Data displayed are mean values from 3 independent colonies. Error bars reflect standard deviation. Indicators of statistical significance: ns *p* > 0.05, * *p* ≤ 0.05, ** *p* ≤ 0.01, *** *p* ≤ 0.001.

An alternative to the use of synthetic POM cycles to increase NADPH, is to express enzymes with NAD^+^ kinase activity. To determine whether overexpression of NAD^+^ kinase activity could improve fatty alcohol biosynthesis, *YEF1*, *UTR1* and a cytosolic version of *POS5*, denoted *POS5c,* were introduced into a high-copy plasmid also harboring ScFAR1 and these constructs were installed in *S. cerevisiae*. Overexpression of *UTR1* (BMY27) or *YEF1* (BMY31) resulted in no significant increase in fatty alcohol production over the control strain BMY12, increasing from 7.23 ± 0.65 mg/L/OD to 8.13 ± 0.77 mg/L/OD (*p* = 0.057) and 6.48 ± 1.34 mg/L/OD (*p* = 0.25) (Fig 6). Overexpression of cytosolic *POS5c* (BMY29) resulted in a significant increase in fatty alcohol titers from 7.23 ± 0.66 mg/L/OD to 11.95 ± 0.82 mg/L/OD **p* *= 0.003 (Fig 6). On their own we found that only *POS5c* was effective at increasing fatty alcohol production and that the increase in production was comparable to expressing the best POM cycle composed of *PYC1, ‘MDH2, sMAE1* on plasmid YCpNC1. In this independent trial, we found that expressing the POM cycle enzymes encoded by YCpNC1 yielded fatty alcohol production of 11.87 ± 0.52 mg/L/OD while expressing Pos5c resulted in 11.95 ± 0.82 mg/L/OD fatty alcohols.

We next sought to investigate whether expression of the NAD^+^ kinases and our synthetic POM cycle could be combined to further increase fatty alcohol production. When combined with YCpNC1 encoding POM cycle enzymes *PYC1, ‘MDH2, and sMAE1*, the overexpression of *YEF1* and *UTR1* had similar effects as they did when expressed with the empty vector control YCplac111 (Fig 6). *UTR1* overexpression combined with YCpNC1 (BMY30) resulted in a trend toward increased fatty alcohol production compared to the control expressing YCpNC1 (BMY13) 12.6 ± 2.23 mg/L/OD vs 11.87 ± 0.52 mg/L/OD *p* = 0.41. The *YEF1* overexpressing strain BMY31 resulted in a slight decrease in fatty alcohol production compared to BMY13. These strains BMY30, BMY31 also displayed the same reduction in final cell density associated with the YCpNC1 carrying control strain (BMY13). The decrease in growth associated with these strains may be due to the fact that both NAD^+^ kinases and the Pyc1 in the POM cycle are ATP-dependent, and therefore effective generation of NADPH to drive fatty alcohol production will occur at the expense of ATP driven processes. Interestingly, combining the overexpression of *POS5c* and YCpNC1 into strain BMY32 did not result in synergistic or even additive effects on fatty alcohol production. Fatty alcohol production in BMY32 (9.58 ± 0.05 mg/L/OD) was less than in the cells expressing either Pos5c alone (11.95 ± 0.82 mg/L/OD, *p* < 0.0001) or the Pyc1, ‘Mdh2, sMae1 POM cycle (BMY13) (11.87 ± 0.05 mg/L/OD, p < 0.0001). The decrease in fatty alcohol production in this strain is also associated with a reduced cell density suggesting extensive metabolic burden or a negative effect caused by the combination of overexpressed gene products.

## Discussion

Re-wiring metabolic pathways in microbial cells to generate cell factories capable of efficient production of desirable bioproducts is a major goal of synthetic biology and metabolic engineering. This is a challenging goal as the endogenous metabolism of cells is optimized for the purpose of biomass accumulation within the organism’s native environment. Altering the flux of carbon and intermediates toward the desired bioproducts typically introduces some degree of metabolic burden and disrupts the flow of intermediates in ways that are not simple to predict.

Many anabolic pathways depend on the availability of NADPH, which can become limiting. In the yeast *S. cerevisiae* two strategies for increasing available NADPH in the cytosol have been investigated. One is expression of enzymatic POM cycles with transhydrogenase-like activity to drive NADPH recycling at the expense of ATP. Alternatively, NAD^+^ kinases can be employed to exploit the more abundant NADH at the expense of ATP. We found that a POM cycle consisting of Pyc1, truncated Mdh2 and cytosolically localized Mae1 (YCpNC1) increased fatty alcohol product titers significantly in *S. cerevisiae*. Although all combinations of the POM cycle enzymes should be capable of increasing NADPH availability, we determined that the other combinations of enzymes tested were unable to increase fatty alcohol production in the context of our experimental strain. Some combinations of POM cycle enzymes lead to unexpected metabolic consequences or increased metabolic burden with little commensurate benefit to redox cofactor biosynthesis. This appears to be especially true of strains overexpressing *PYC2*, where both reduced cell growth and decreased fatty alcohol production were observed. Why Pyc1 out performs Pyc2 in these cycles is not clear. It may simply be that Pyc1 accumulates to higher levels than Pyc2 or that the relative abundance of Pyc1 and the Mdh expressed results in more stoichiometric expression. However, we cannot discount the possibility that the functional differences between the isoforms in this context is due to allosteric regulation by cellular metabolites or other inherent differences between the protein isoforms.

In the context of utilizing a synthetic POM cycle to generate NADPH, the stoichiometry of the enzymes is likely to be as important as the total activity for optimal function. High pyruvate carboxylase activity may not be optimal for operation of the POM cycle unless it can be matched by the activity of the malate dehydrogenase and malic enzyme. The POM cycle employing Pyc1 ‘Mdh2 sMae1 yielded higher fatty alcohol production than did the Pyc1 ‘Mdh1, sMae1 cycle. This is consistent with the notion that Mdh2 is more effective in the context of this cycle. The coding sequences for the two malate dehydrogenase isoforms were modified to provide stable cytosolic expression in glucose and showed comparable protein abundance in actively proliferating cells; however, Mdh2 is native to the cytosol whereas Mdh1 normally functions in the mitochondria with a different redox environment. It may also be significant that Mdh2 displays a higher affinity for NADH than does Mdh1, which may make it more effective in driving the oxaloacetate to malate reaction [25]. Another isoform of malate dehydrogenase, the peroxisome associated Mdh3 has also been employed in a POM cycle [17]. However, Mdh3 has a significantly lower affinity for oxaloacetate than does Mdh2 suggesting it might not be the best enzyme to drive a POM cycle for NADPH synthesis. Additionally other factors may influence the accumulation and activity of Mdh isoforms in the cytosol as over production of a cytosolically targeted Mdh3 yields a 2-fold increase in MDH activity compared to ‘Mdh2 that gave a 6–8 fold increase [26].

Previous reports on application of synthetic POM cycles for metabolic engineering in *S. cerevisiae* all employ the endogenous Pyc2 with Mdh2 and Mae1. They have been reported to counter the redox imbalance of a heterologous xylose fermentation pathway [16] and for the production of isobutanol [15]. However, while the expression of the cycle in xylose fermenting conditions was reported to increase the rate of xylose consumption, ultimately it resulted in decreased ethanol production [16]. When implemented in isobutanol producing strains, a version of the cycle expressing a truncated Mae1 (sMae1p) increased isobutanol production in a strain overexpressing the Ehrlich pathway and *ILV2* but had no effect on isobutanol titers in other strains [15]. While the efficacy of these POM cycles is not immediately clear [12,14–16], it is possible that they may be improved by overexpressing Pyc1 rather than Pyc2 as we have shown that enzyme combinations including Pyc1, when combined with Mdh2, outperform those containing Pyc2. Adding support to this argument, a recent report converting *S. cerevisiae* to lipogenic metabolism utilized a Pyc1 containing POM cycle with success, however alternate combinations were not reported [17].

Several of the POM cycle enzyme combinations failed to increase fatty alcohol products and reduced cellular growth. Even the POM cycle (Pyc1, ‘Mdh2, sMAE1) that did increase fatty alcohol production lead to a perturbation of several metabolic intermediates that we could detect. Fumarate and aspartate were significantly reduced while several intermediates in amino acid metabolic pathways were increased. Aspartic acid has a close relationship with central carbon metabolism, it can be produced from OAA by transamination and can similarly act as a precursor for OAA production [49]. In *S. cerevisiae*, aspartate is primarily produced by transamination of OAA. The reduction in aspartate in the POM cycle expressing strain may reflect a diversion of aspartate to OAA to meet the elevated requirement for OAA required to maintain the TCA cycle activity in addition to the increased cytosolic POM cycle activity. Aspartate also acts as a precursor in the synthesis of arginine, methionine, isoleucine, asparagine and threonine [4]. It may be significant that we observed a reduction in methionine, asparagine and threonine in the POM cycle strain. The reduction in abundance of these amino acids did not reach statistical significance but this may have been influenced by the presence of exogenous amino acids present in the culture medium. Analysis of strains cultured in fully minimal medium, lacking amino acids would more fully elucidate the effects of the POM cycle on amino acid metabolic pathways.

Fumarate is an intermediate in the TCA cycle produced by the oxidation of succinate. It can also be produced from arginino-succinate by Arg4 in the urea cycle and synthesis of arginine [50]. The reduction in fumarate observed in POM cycle expressing strains could indicate that the POM cycle is drawing pyruvate and intermediates from the tricarboxylic acid cycle (TCA), possibly owing to disparity in reaction rates of the POM cycle enzymes. This may be tolerable under the ideal laboratory culture conditions but is likely not optimal. This idea is consistent with the increase in citrulline, and decreased aspartic acid we observed as the cells may be using these intermediates to synthesize arginino-succinate in the urea cycle as a way to replenish fumarate. Several intermediates that were elevated in the POM cycle expression strain can be generated by amino acids degradation processes. Citrulline is an intermediate in arginine synthesis but can also be produced from arginine in an NADPH dependent reaction [51]. Our metabolomic analysis also identified increases in 5-hydroxyectoine, which can be derived from aspartic acid in an NADPH dependent reaction and saccharopine, a lysine synthesis and degradation intermediate that can be derived from lysine in an NADPH dependent reaction [52]. It is currently unclear whether the changes in metabolite abundance in the POM cycle expressing strain are driven directly by over expression of one or more of the POM cycle enzymes or are part of the cellular response to alterations in the redox cofactor balance induced by the POM cycle.

Central carbon metabolism and the balance of NADPH/NADP^+^ redox cofactors are tightly linked with amino acid metabolism. NADPH is a driver of the lysine, proline and threonine biosynthetic pathways and amino acids can be used as precursors for intermediates in TCA cycle, in turn the TCA cycle can provide precursors for amino acid biosynthesis. This allows flexibility to replenish intermediates in the TCA and to provide amino acids if needed. This relationship can potentially influence the entire metabolic network since NADPH influences flux through most anabolic pathways. Either an excess or a deficiency in NADPH has negative effects on cell growth rate and carbon utilization in *E. coli* highlighting the need to balance NADPH availability with cellular needs [53]. Elevating NADPH availability is effective in driving flux through engineered anabolic pathways including lipid and fatty alcohol production but excess NAPDH, beyond what can be utilized to meet existing cellular needs, has the potential to yield reductive stress [54]. This effect is not well studied in yeast but excess NADPH can inhibit Zwf1 and Gnd1 reducing flux through the pentose phosphate pathway with a commensurate increase in flux of carbon through glycolysis [55,56]. This alteration in pathway flux has the potential to change the cellular concentrations of a wide range of intermediates. Another potential effect of altering NADPH/NADP^+^ balance is that excess NADPH could drive reactions including amino acid biosynthesis leading to an increase in amino acid synthesis and degradation intermediates [57].

The catalytic activity and abundance of the POM cycle enzymes are not the only factors influencing their effectiveness in the context of NADPH regeneration. The cellular localization, spatial organization and native protein-protein interactions of the enzymes may have substantial influence on which isoforms will work most effectively together. Physical interactions have been demonstrated between Pyc1 - Pyc2, Pyc1 - Mdh1 and Pyc2 - Mdh1 as well as between Mdh1 - Mdh2 and Mdh1 - Mdh3 [58,59]. It is unclear whether these interactions are all biologically relevant in the endogenous context but they do suggest that Mdh1 might be capable of functioning effectively with Pyc1 or Pyc2 in some contexts. Mdh2 has been demonstrated to self-interact and this may have implications for the ability generate high local concentration of product [60]. Mdh2 is most active in cells growing on nonfermentable 2-carbon compounds and takes part in the glyoxylate cycle and gluconeogenesis. Our study made use of a truncated variant of Mdh2 demonstrated to have increased stability and catalytic activity in cells cultured in glucose containing medium [61]. In addition to stabilizing the enzyme the amino-terminal truncation also disrupts its physical interactions with partners Pck1 and Fbp1, enzymes that it functions with in gluconeogenesis [62]. Disruption of those interactions may free the cytosolic Mdh2 to work more effectively with Pyc1 in the context of the POM cycle tested in our strains. In this regard installing a POM cycle composed of fully orthogonal enzymes might be more effective without the need to overcome endogenous physical interactions with the native enzymes. Synthetic biology approaches that could offer alternative strategies to increase the effectiveness of the POM cycle enzymes would be to create a scaffold to sequester the POM cycle enzymes together in a cytosolic location or test the potential of expressing enzyme fusions separated by flexible linkers to maintain the enzymes in close proximity to one another.

The ability of the cytosolically localized NAD^+^ kinase Pos5 to improve fatty alcohol synthesis is consistent with the effectiveness of this enzyme in yielding improved performance of other engineered metabolic pathways [32,34]. Pos5c was the only NAD^+^ kinase to show a significant increase in fatty alcohol production possibly reflecting that Pos5 phosphorylates NADH preferentially while Yef1 and Utr1 display activity towards both NAD^+^ and NADH. It is less clear why combining the POM cycle and Pos5c fails to achieve any additive improvement considering that they draw on different substrates to yield NADPH. This may reflect an excess metabolic burden imposed on the cells or the combined activities of these pathways may be disruptive toward functions of each other in ways we have failed to predict.

One POM cycle tested in this study was effective in increasing the production of fatty alcohol. The POM cycle also influenced the abundance of intermediates in central carbon metabolism and amino acid metabolism. Thus, from a metabolic engineering perspective, effective design and implementation of a POM cycle to increase NADPH availability will need to consider the effects of that cycle on the entire cellular metabolic network. A more comprehensive metabolomic analysis covering a higher fraction of the intermediates in amino acid, purine and pyrimidine synthesis and degradation pathways will be required to gain a firmer understanding of the consequence of this POM cycle to cellular metabolism. Those data would allow for an improved estimation of the abundance of allosteric regulators of POM cycle enzyme activity to guide enzyme selection. This study employed constitutive expression of the POM cycle enzymes. While this is a practical strategy from the bioproduction perspective it makes direct effects on difficult to separate from secondary effects. Further investigations into the effects of POM cycle expression could be performed using inducible POM cycle enzymes to allow observations to be made immediately following induction and monitor metabolites to determine how the cells metabolic network adapts to the acute increased in NADPH availability. Proteomic analysis of strains expressing POM cycle enzymes would also be useful to provide data on enzyme stoichiometry and identify changes in the abundance of enzymes in other metabolic pathways affected by the POM cycle. Future attempts to optimize NADPH production in the cytoplasm will need to work toward balancing catalytic rates of the enzymes and maintaining local proximity to minimize side reactions.

This investigation has revealed that not all POM cycles will be suitable for promoting NADPH regeneration to drive anabolic pathways. We have identified one set of POM cycle enzymes proven beneficial for production of fatty alcohols and while we can speculate on why the other combinations are less successful it is clear that more extensive testing is required to determine what parameters need to be optimized. More extensive metabolomic analysis and potentially metabolic model simulations will benefit our ability to apply this manipulation to achieve predictable improvements in bioproduct synthesis.

## Supporting information

S1 Fig Production of Fatty alcohols by initial candidate colonies of FAS overexpressing strains containing synthetic POM cycles. (A) Average production from 7 independent candidate colonies from two unique transformation events of each strain are shown with standard deviation (B) The individual production of the candidate colonies of BMY13 are shown in. Data shown here is not adjusted for changes in OD associated with the strains.
(DOCX)

S2 Fig Production of fatty alcohols by high producing strains. Fatty alcohol concentrations (mg/L) produced by strains carrying synthetic POM cycles and the corresponding culture OD_600_ measurements after 72 hours growth. Bars indicate mean Fatty acid concentrations (mg/L). Culture density is indicated by the unfilled circles. Error bars indicate standard deviation derived from 3 biological replicates.
(DOCX)

S3 Fig Overexpression of individual genes from any of the POM cycles do not increase fatty alcohol production. The effect of overexpression of the enzymes sMae1(BMY17), ‘Mdh1 (BMY18), ‘Mdh2 (BMY19), Pyc1(BMY20), and Pyc2 (BMY21), fatty alcohol production as compared to the empty vector expressing control strain (BMY12). (n = 3) Error displayed as standard deviation.
(DOCX)

S1 Table Oligonucleotide primers used in this study. (DOCX)

S2 Table Metabolites positively identified. (PDF)

S3 Table The NC1 POM cycle influences amino acid metabolism. A pathway impact table based on metabolite differences observed between *S. cerevisiae* strains harboring an empty vector of the NC1 POM cycle.
(DOCX)
