# Supplementary material for: Evaluating the effects of synthetic POM cycles and NAD+ kinase expression on fatty alcohol production in Saccharomyces cerevisiae
Source: PLoS One. 2025 Sep 29;20(9):e0333299. doi: 10.1371/journal.pone.0333299 (PMC12478946; doi:10.1371/journal.pone.0333299)
Supplement: S1 Fig — (A) Average production from 7 independent candidate colonies from two unique transformation events of each strain are shown with standard deviation (B) The individual production of the candidate colonies of BMY13 are shown in. Data shown here is not adjusted for changes in OD associated with the strains. (DOCX) [file pone.0333299.s001.docx]

**
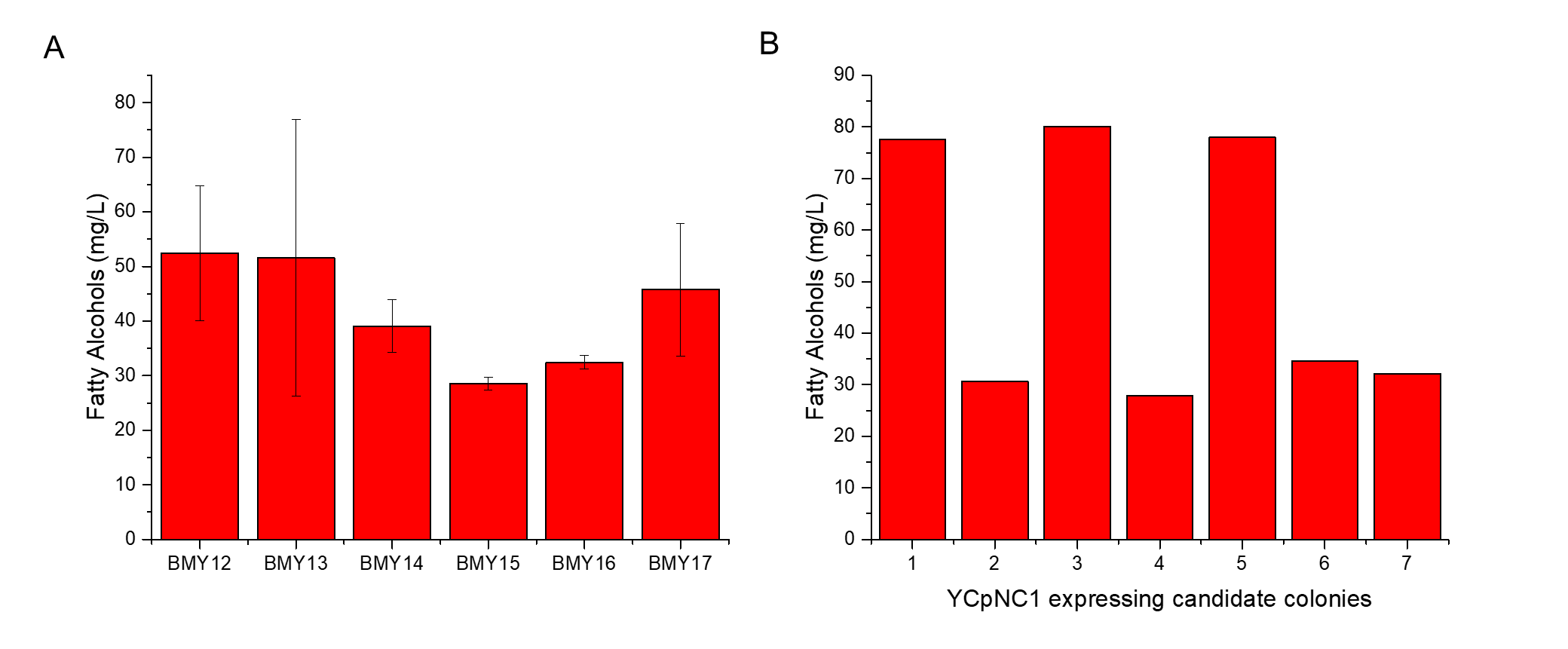
**

**S1 Fig.** Production of Fatty alcohols by initial candidate colonies of FAS overexpressing strains containing synthetic POM cycles. (A) Average production from 7 independent candidate colonies from two unique transformation events of each strain are shown with standard deviation (B) The individual production of the candidate colonies of BMY13 are shown in. Data shown are not adjusted for variations in OD associated with the cultures.
