# Supplementary material for: Evaluating the effects of synthetic POM cycles and NAD+ kinase expression on fatty alcohol production in Saccharomyces cerevisiae
Source: PLoS One. 2025 Sep 29;20(9):e0333299. doi: 10.1371/journal.pone.0333299 (PMC12478946; doi:10.1371/journal.pone.0333299)
Supplement: S2 Fig — Fatty alcohol concentrations (mg/L) produced by strains carrying synthetic POM cycles and the corresponding culture OD600 measurements after 72 hours growth. Bars indicate mean Fatty acid concentrations (mg/L). Culture density is indicated by the unfilled circles. Error bars indicate standard deviation derived from 3 biological replicates. (DOCX) [file pone.0333299.s002.docx]

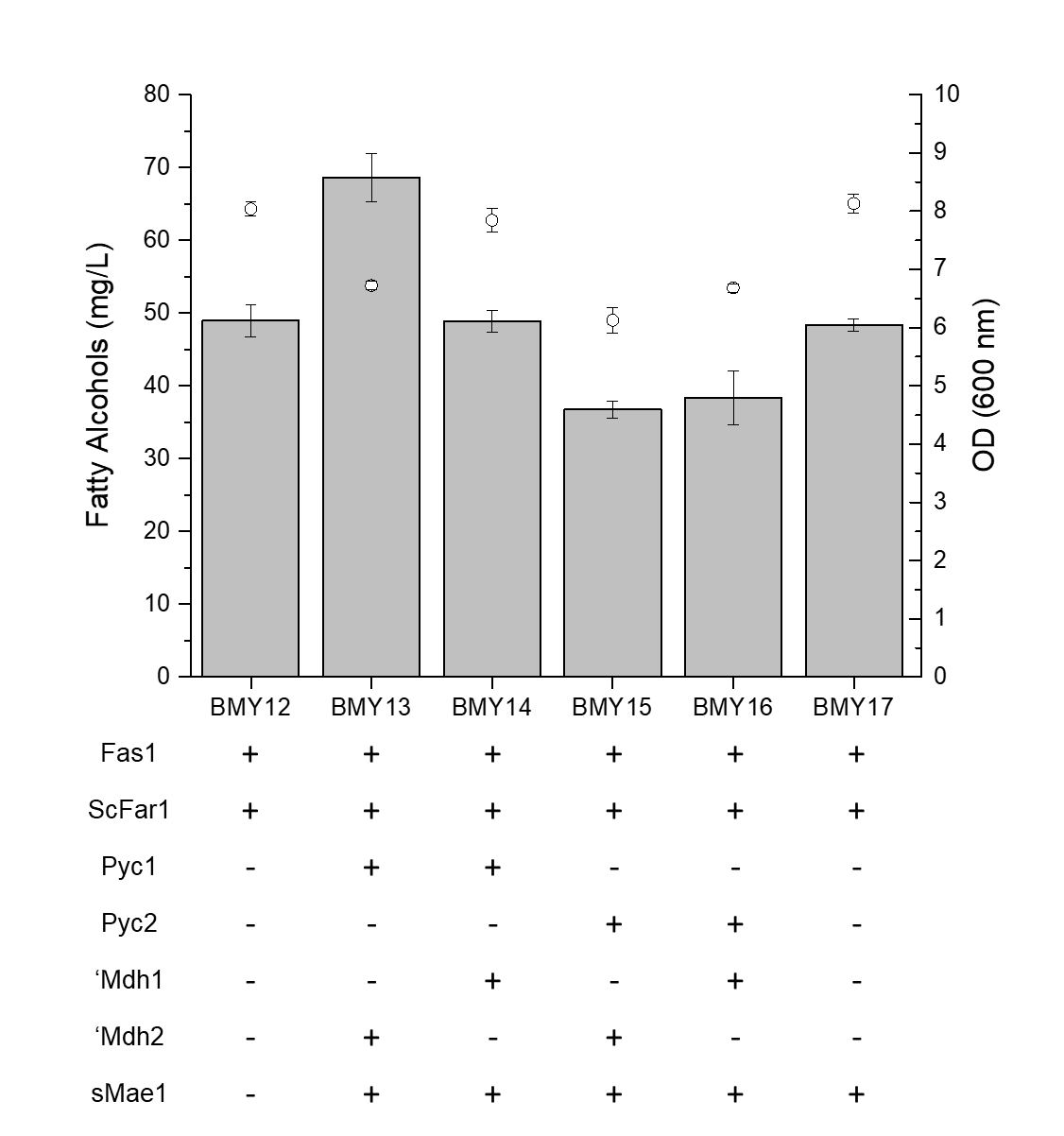


**S2 Fig.** Production of Fatty alcohols by high producing colonies of FAS overexpressing strains containing synthetic POM cycles and the corresponding culture OD_600_ measurements after 72 hours growth. Bars indicate mean Fatty acid concentrations (mg/L). Culture density is indicated by the unfilled circles. Error bars indicate standard deviation derived from 3 biological replicates.
