# Supplementary material for: Evaluating the effects of synthetic POM cycles and NAD+ kinase expression on fatty alcohol production in Saccharomyces cerevisiae
Source: PLoS One. 2025 Sep 29;20(9):e0333299. doi: 10.1371/journal.pone.0333299 (PMC12478946; doi:10.1371/journal.pone.0333299)
Supplement: S3 Fig — The effect of overexpression of the enzymes sMae1(BMY17), ‘Mdh1 (BMY18), ‘Mdh2 (BMY19), Pyc1(BMY20), and Pyc2 (BMY21), fatty alcohol production as compared to the empty vector expressing control strain (BMY12). (n = 3) Error displayed as standard deviation. (DOCX) [file pone.0333299.s003.docx]

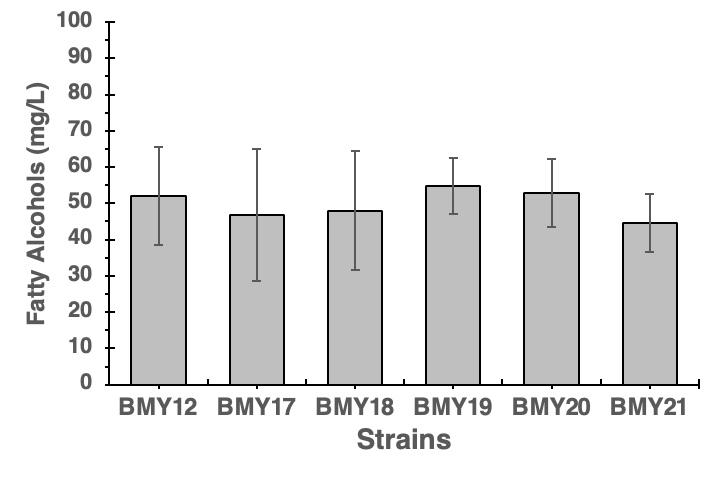


**S3 Fig.** **Overexpression of individual genes from any of the POM cycles do not increase fatty alcohol production.** The effect of overexpression of the enzymes sMae1(BMY17), ‘Mdh1 (BMY18), ‘Mdh2 (BMY19), Pyc1(BMY20), and Pyc2 (BMY21), fatty alcohol production as compared to the empty vector expressing control strain (BMY12). (n=3) Error displayed as standard deviation.
