# Supplementary material for: Evaluating the effects of synthetic POM cycles and NAD+ kinase expression on fatty alcohol production in Saccharomyces cerevisiae
Source: PLoS One. 2025 Sep 29;20(9):e0333299. doi: 10.1371/journal.pone.0333299 (PMC12478946; doi:10.1371/journal.pone.0333299)
Supplement: S1 Table — (DOCX) [file pone.0333299.s004.docx]

**Table S1.** Oligonucleotide sequences used for plasmid construction

| **Name** | **Sequence** | **Use** |
| --- | --- | --- |
| 5’-HXT-V5 | CCTGCAGGTCGACTCTAGAGCCACTACTTCTCGTAGCAAC | Amplify pHXT7 |
| HXTa3rs | CTCCCGGGATATCCTAGGTTGTTTTTGATTAAAATTAAAAAAACTTTTT |  |
| FBAa5rs | CAACCTAGGATATCCCGGGAGTTGATGGATCCAACTGGC | Amplify pFBA1 |
| FBAa3 | TTTGAATATGTATTACTTGGTTATG |  |
| P3-O3 | TAACCAAGTAATACATATTCAAAATGTGGCCTATTCAGCAATCGCG | Amplify sMAE1 |
| 3’-MAE1noter | CCAGTGAATTCGAGTCTCGGTACCCCTACAATTGGTTGGTGTGCA |  |
| HXT*-P1 | AAAGTTTTTTTAATTTTAATCAAAAACAACATGTCGCAAAGAAAATTCGCC | Amplify PYC1 +250ds |
| PYC1-3 | AAGATGTTTCGCTAAAAGATGC |  |
| HXT*-P2 | AAAGTTTTTTTAATTTTAATCAAAAACAACATGAGCAGTAGTCAAGAAATTGG | Amplify PYC2  +250ds |
| PYC2-3 | TAAATAGAGTAGTTAACGTACTTG |  |
| O1-P2 | CATCTTTTAGCGAAACATCTTATCACACCCAATCCCCCAC | Amplify TEF1p |
| O4-P2 | AGTACGTTAACTACTCTATTTAATCACACCCAATCCCCCAC |  |
| TEFa3 | TTTGTAATTAAAACTTAGATTAGATT |  |
| P2-O2 | TCTAATCTAAGTTTTAATTACAAAATGTCGTTAAAAATTGCCATTT | Amplify ‘MDH2+250ds |
| Mdh2-F1 | GCCAGTTCCATCCATCAACTCCCAGTAAAGAGTTGTACATCAGGT |  |
| P2-O5 | TCTAATCTAAGTTTTAATTACAAAATGTATAAAGTGACTGTTTTGG | Amplify ‘MDH1+250ds |
| Mdh1-F1 | GCCAGTTGGATCCATCAACTCCCAATCATTTTTGTTTTTCCTCGTC |  |
| MAE-C5 | AATTGTCGCCACTAAGAGAGG | Amplify C-term of MAE1 |
| MAE-C3 | GCGAAATAGATTATAATGCCTACAATTGGTTGGTGTGCAC |  |
| CDCt5 | GTGCACACCAACCAATTGTAGGCATTATAATGTATTTCGC | Amplify CDC28 terminator |
| CDCt3 | GGTGAGCGAATTCTTCATTTTCTTTGATAATACGACC |  |
| SalI-FBA1p | CCCGGTCGACAGTTGATGGATCCAACTG | Used with MAE-C3 to amplify FBA1p-MAE |
| 5VH HXT7 | GCATGCCTGCAGGTCGACTCTAGAGGATCCCCACTACTTCTCGTAGGAAC | Used to clone single enzyme controls with and without myc tag |
| 3 PYC1 MYC | CAATCTTCTTCAGAAATCAATTTTTGTTCTGCCTTAGTTTCAACAGGAACTTGG |  |
| 5 MYC PYC1 TER | GAACAAAAATTGATTTCTGAAGAAGATTTGTGAACCGGTTAGTTCTCATTTATAATG |  |
| 3VH PYC1 TER | CGACGGCCAGTGAATTCGAGCTCGGTACCCAAGATGTTTCGCTAAAAGATGC |  |
| 3 PYC2-MYC | CAATCTTCTTCAGAAATCAATTTTTGTTCCTTTTTTTGGGATGGGGGTAG |  |
| 5 MYC PYC2TER | GAACAAAAATTGATTTCTGAAGAAGATTTGTAATTTTTACTCGTTAATTATATTTTATGA |  |
| 3VH PYC2 TER | CGACGGCCAGTGAATTCGAGCTCGGTACCCTAAATAGAGTAGTTAACGTACTTG |  |
| 5VH TEF1 | GCATGCCTGCAGGTCGACTCTAGAGGATCCATCACACCCAATCCCCCAC |  |
| 3 MDH1-MYC | CAAATCTTCTTCAGAAATCAATTTTTGTTCTTTACTAGCAACAAAGTTGACACC |  |
| 3VH MDH1 ter | CGACGGCCAGTGAATTCGAGCTCGGTACCCTCATTTTTGTTTTTCCTCGTCTACG |  |
| 3 MDH2 MYC NEW | CAAATCTTCTTCAGAAATCAATTTTTGTTCAGATGATGCAGATCTCGATGC |  |
| 5 MYC MDH2TER | GAACAAAAATTGATTTCTGAAGAAGATTTGTAATGAGCATCGGACCGAAGCA |  |
| 3VH MDH2TER | CGACGGCCAGTGAATTCGAGCTCGGTACCCAGTAAAGAGTTGTACATCAGGTAAG |  |
| UTR1 5 NEW | GCGTCTAGAAAAAAAATGAAGGAGAATGACATGAAT | Used to clone NAD Kinases |
| UTR1 3 | CCGCAACTGCAGAAGCACTAGTAATTACCTACTTGAAAAAAGCC |  |
| YEF1 5 | GCGTCATCTAGAAAAAAAATGAAAACTGATAGATTAC |  |
| YEF1 3 NEW | TCCCTGCAGGCACTAGTAACCTTATCATATGGTTAA |  |
| POS5C 5 NEW | CGCCCTAGGAAAAAAATGAGTACGTTGGATTCACATTC |  |
| 3’ POS5 | ATACTGCAGACTAGTCTTCTTGTTAGGCATGTCTTCCC |  |
| PYC1 F1 | GGT GTC AAG ACC AAC ATT CCC TTC C | RT PCR |
| PYC1 R1 | CAT TGC CCT GAG CAT CGT GC | RT PCR |
| PYC1 F2 | TTT CGA GGC TGA CTT GAA GGG C | RT PCR |
| PYC1 R2 | AAA TTG TGC CAG ATC ACC AAC GAC C | RT PCR |
| PYC2 F1 | GCT ACT ACT CTC GCC TCA CTA CG | RT PCR |
| PYC2 R1 | AAC CGT TAA CTG CCA AGT CTG CC | RT PCR |
| PYC2 F2 | CGT GAA TTA GAT GCA TAC TGG GCC G | RT PCR |
| PYC2 R2 | CAA AAA AGT CCA TAA CAG AGT CAG GAA AGT CC | RT PCR |
| MDH1 F1 | CAA AGA ATG GTG CTG GCT CTG CTA CG | RT PCR |
| MDH1 R1 | ATG GAT CTT TTC AAT ACC ATC TGG GCC | RT PCR |
| MDH2 F1 | TGT AAT TGG CGG GCA TTC TGG C | RT PCR |
| MDH2 R1 | TTC CAT GGA TCT GCT CAA TGT TAC CC | RT PCR |
| MAE1 F1 | GGG AAT TGG TGA CCA AGG TAT CGG | RT PCR |
| MAE1 R1 | CCC ATG TAC AAT TCG TCA CGG GC | RT PCR |
| PYC2D5 | ATTACTATATTGCAAAATAAAGGACAGTTACTAGGAGAGAAAATAAGGGACATAGAGAAC***AAGCTTCGTACGCTGCAGG*** | delete PYC2 5' |
| PYC2D3 | CGCCATATATAGTACAGCTAGTATTTTCAGATGTCATAAAATATAATTAACGAGTAAAAA***CGACTCACTATAGGGAGACC*** | delete PYC2 3' |
