# Supplementary material for: Evaluating the effects of synthetic POM cycles and NAD+ kinase expression on fatty alcohol production in Saccharomyces cerevisiae
Source: PLoS One. 2025 Sep 29;20(9):e0333299. doi: 10.1371/journal.pone.0333299 (PMC12478946; doi:10.1371/journal.pone.0333299)
Supplement: S2 Table — (PDF) [file pone.0333299.s005.pdf]

Compound	Fold Change	p-value	q-value	Significance
Acetic Acid	0.772026752	0.089981165	0.285432214	Not Significant
Acrylic Acid	1.190941039	0.047579399	0.240198448	Not Significant
Butanoic Acid / Isobutyric Acid	1.007497657	0.939568445	0.69345528	Not Significant
Lauric Acid	0.645661157	0.387252283	0.498084974	Not Significant
8-Methyl-6-nonenoic acid	1.226905515	0.104222175	0.297610096	Not Significant
Crotonic acid	1.458631663	0.075254764	0.270139896	Not Significant
Pelargonic acid	1.024684102	0.421411398	0.511740907	Not Significant
Isomer 1 of Acetic Acid	0.913580247	0.545722121	0.570276153	Not Significant
Isomer 1 of Acrylic Acid	1.015042511	0.845385795	0.669520867	Not Significant
6-Oxo-1,4,5,6-tetrahydronicotinic acid	1.221146953	0.14143622	0.332575052	Not Significant
4-Hydroxyproline	1.024486226	0.921623083	0.690347303	Not Significant
A-Aminobutyric Acid	1.073229292	0.377066156	0.491726898	Not Significant
Acetylglycine	1.128240581	0.561692585	0.575386502	Not Significant
Alanine	1.201553254	0.002911572	0.148733377	Not Significant
Alloisoleucine	0.938195303	0.653398811	0.598764957	Not Significant
Arginine	1.030175439	0.79888865	0.655879433	Not Significant
Asparagine	0.680398492	0.044774359	0.239384024	Not Significant
Aspartic Acid	0.43049543	0.000376242	0.130023696	Significant Decrease
Citrulline	2.334713187	0.000937354	0.136212565	Significant Increase
Cystine	0.878081279	0.47193769	0.532933308	Not Significant
Glutamic Acid	0.997539543	0.965330284	0.70199957	Not Significant
Glutathione	0.633864947	0.057203595	0.24508997	Not Significant
Histidine	0.934123847	0.486890682	0.541266793	Not Significant
Homoserine	0.722604705	0.01955649	0.189453493	Not Significant
Kynurenine	0.766063631	0.238040758	0.406731059	Not Significant
Leucine	0.977616646	0.822884278	0.663572912	Not Significant
Lysine	1.251472116	0.043063596	0.236691698	Not Significant
Methionine	0.870646766	0.145357826	0.334756842	Not Significant
Methionine Sulfoxide	1.154382105	0.321917919	0.4627078	Not Significant
N-Methyl aspartic acid	0.966835871	0.393205895	0.501620689	Not Significant
N6-Acetyl-Lysine	1.256925996	0.037968822	0.228538487	Not Significant
Oxidized glutathione	0.98633678	0.830115767	0.665423608	Not Significant
Phenylalanine	0.914249283	0.436039882	0.518363083	Not Significant
Pipecollinic Acid	1.205513784	0.056957161	0.244746612	Not Significant
Proline	0.958639093	0.417102067	0.510778308	Not Significant
Pyroglutamic Acid	0.816136773	0.055737599	0.24299145	Not Significant
S-Adenosylhomocysteine	0.804871591	0.345252384	0.475687508	Not Significant
Saccharopine	2.554776512	0.040139537	0.232895172	Significant Increase
Serine	0.802667475	0.094813203	0.290837295	Not Significant
Symmetric dimethylarginine	1.075079309	0.694845522	0.616945492	Not Significant
Threonine	0.602526203	0.068006986	0.258168326	Not Significant
Tryptophan	1.031818182	0.852319068	0.671511874	Not Significant
Tyrosine	1.062776025	0.711045669	0.622572083	Not Significant
Valine	1.079760718	0.232964721	0.403460817	Not Significant
Isomer 1 of Glutamic Acid	1.066258552	0.359570677	0.484939943	Not Significant
Isomer 1 of Homoserine	0.932373619	0.694047788	0.616741892	Not Significant
Isomer 1 of N-Methyl aspartic acid	1.100817439	0.344360488	0.475200879	Not Significant
Isomer 1 of Pipecollinic Acid	1.092413236	0.501769374	0.548810252	Not Significant

(S)-2-Azetidinecarboxylic acid	0.664658635	0.046650793	0.240081416	Significant Decrease
2-(3-Carboxy-3-aminopropyl)-L-histidine	1.017660911	0.871247909	0.677452733	Not Significant
2-Amino-5-oxohexanoic acid	1.119609439	0.504843124	0.550230828	Not Significant
3-Amino-2-piperidone	0.910657307	0.637014165	0.59365732	Not Significant
3-Cyano-L-alanine	0.725131701	0.026974526	0.211124116	Not Significant
3-Sulfino-L-alanine	0.838729571	0.058927179	0.24795085	Not Significant
4-Chloro-L-lysine	1.242139649	0.078668012	0.271830551	Not Significant
4-Hydroxy-4-methylglutamic acid	0.771033654	0.018883169	0.185533142	Not Significant
4-Hydroxy-L-threonine / (+)-threo-2-Amino-3,4-dihydroxybutanoic acid	1.66983017	0.116272326	0.307321494	Not Significant
4-Methyl-L-glutamic acid	0.254573171	0.021142091	0.196902734	Significant Decrease
4-Methylene-L-glutamic acid	1.136290062	0.078428938	0.271830551	Not Significant
4-Oxoproline	0.983196159	0.611519349	0.58794727	Not Significant
5-Hydroxyectoine	1.887267237	0.006986236	0.156115145	Significant Increase
5-Hydroxykynurenine	1.468461822	0.138657011	0.330785934	Not Significant
5-Methoxytryptophan	1.056120194	0.624823217	0.592081389	Not Significant
Betalamic acid	1.214496418	0.334261132	0.469784104	Not Significant
Bis-gamma-glutamylcystine	1.260854817	0.252797745	0.417799777	Not Significant
Cycloalliin	0.741935484	0.069081474	0.258906388	Not Significant
Cystathionine sulfoxide	1.704785894	0.291946134	0.447719513	Not Significant
D-Lysopine	1.856756757	0.001396603	0.136212565	Significant Increase
DL-2-Aminooctanoic acid	1.323225362	0.059944782	0.247999301	Not Significant
Epsilon-(gamma-Glutamyl)-lysine	1.175016057	0.261930458	0.422791013	Not Significant
Histidinyl-Gamma-glutamic acid	0.942213516	0.681772434	0.610313229	Not Significant
Homocysteinesulfinic acid	0.941798942	0.345977505	0.475773882	Not Significant
Hydroxyethyl glycine	0.620182013	0.12789606	0.323090163	Not Significant
Hypoglycin B	0.710587147	0.430151729	0.514569599	Not Significant
Isoalliin	0.751925008	0.090950062	0.287280399	Not Significant
L-2-Amino-3-oxobutanoic acid	0.959542656	0.773502142	0.648570189	Not Significant
L-2-Amino-5-hydroxypentanoic acid	1.106336261	0.100233055	0.29392666	Not Significant
L-2-Amino-6-oxoheptanedioic acid	1.446071254	0.288504805	0.446215008	Not Significant
L-2-Aminoadipate 6-semialdehyde / Allysine	1.155949367	0.04554703	0.240081416	Not Significant
L-Aspartate 4-semialdehyde	1.289764359	0.307112715	0.456462048	Not Significant
L-Cysteic acid	0.926796953	0.17716072	0.360636737	Not Significant
L-Glutamine / D-Glutamine	1.008454515	0.926636664	0.690759726	Not Significant
L-Homocysteine	0.923754659	0.677968182	0.609188801	Not Significant
L-Hypoglycin A	1.190311419	0.131547094	0.328480152	Not Significant
L-N-Carboxymethylserine	0.521424071	0.059461385	0.247999301	Not Significant
L-Norleucine	1.074633124	0.726145563	0.628534452	Not Significant
L-Ornithine / D-Ornithine	1.598284222	0.061595775	0.249978299	Not Significant
L-Propargylglycine	1.154605263	0.19731462	0.383323836	Not Significant
L-Targinine	1.037649743	0.770607216	0.647143056	Not Significant
L-Thyronine	1.348587346	0.238020881	0.406731059	Not Significant
L-erythro-4-Hydroxyglutamic acid / 4-Hydroxy-L-glutamic acid	0.615539568	0.038011344	0.228538487	Significant Decrease
L-threo-3-Methylaspartic acid	0.905589343	0.102236778	0.296827663	Not Significant
L-trans-5-Hydroxy-2-piperidinecarboxylic acid	0.981200289	0.938305931	0.69345528	Not Significant
Lysyl-Gamma-glutamic acid	1.038343033	0.695340898	0.61705703	Not Significant
N(gamma)-Acetyldiaminobutyric acid	1.333333333	0.547675347	0.570276153	Not Significant
N-Acetyl-L-Methionine	0.753605769	0.028543868	0.211124116	Not Significant
N-Acetyl-L-Tyrosine	0.769430725	0.038360449	0.228538487	Not Significant

N-Acetyl-L-aspartic acid	1.551579749	0.155124482	0.342093623	Not Significant
N-Acetyl-L-glutamic acid	1.130388693	0.012539577	0.166956837	Not Significant
N-Formyl-L-aspartic acid	1.174224344	0.112114266	0.306408006	Not Significant
N-Methyl-L-glutamic acid	0.44364564	0.014109596	0.171457226	Significant Decrease
N2'-Acetyl-L-Cystathionine	1.078358209	0.554212098	0.572685835	Not Significant
N5-Acetyl-L-Ornithine	0.783791866	0.246061144	0.412268944	Not Significant
Ne,Ne dimethyllysine	0.959824047	0.795481375	0.654651054	Not Significant
O-Acetyl-L-homoserine	0.780012571	0.115208587	0.307279086	Not Significant
Ophthalmic acid	1.299809524	0.241122442	0.410059325	Not Significant
Oxypinnatanine	0.989637306	0.893330881	0.684508479	Not Significant
Penmacric acid	3.466179411	0.099263256	0.292664763	Not Significant
Phenylacetylglycine	1.029221882	0.811046256	0.659124848	Not Significant
S-Glutathionyl-L-cysteine - 2 tags / Cysteineglutathione disulfide	1.321806347	0.109381737	0.301982658	Not Significant
S-Sulfanylglutathione	1.077805332	0.696357432	0.61705703	Not Significant
Valyl-Gamma-glutamic acid	1.003036053	0.970561098	0.702378675	Not Significant
Vinylacetylglycine	1.05284416	0.138187671	0.330785934	Not Significant
beta-Alanyl-N(pi)-methyl-L-histidine	0.961895613	0.783670806	0.651058824	Not Significant
gamma-Glutamylleucine	0.991933315	0.954184673	0.69763502	Not Significant
gamma-Glutamylphenylalanine	1.039458414	0.725575374	0.628291525	Not Significant
gamma-Glutamyltyrosine	1.094819466	0.504739281	0.550230828	Not Significant
Isomer 1 of 2-(3-Carboxy-3-aminopropyl)-L-histidine	0.868380303	0.098316741	0.292657514	Not Significant
Isomer 1 of 3-Cyano-L-alanine	0.921280854	0.291328747	0.447405082	Not Significant
Isomer 1 of 4-Methylene-L-glutamic acid	1.26344086	0.19701022	0.383323836	Not Significant
Isomer 1 of Acetylglycine	0.776744186	0.310280503	0.458033123	Not Significant
Isomer 1 of Arginine	1.092787378	0.362219636	0.486515081	Not Significant
Isomer 1 of Glutathione	0.892248311	0.198418749	0.383371616	Not Significant
Isomer 1 of Histidine	0.996963842	0.970509713	0.702378675	Not Significant
Isomer 1 of Histidinyl-Gamma-glutamic acid	0.973067174	0.810480786	0.659124848	Not Significant
Isomer 1 of L-Hypoglycin A	1.177516576	0.011080369	0.166956837	Not Significant
Isomer 1 of L-Ornithine / D-Ornithine	0.716466739	0.032747125	0.219607297	Not Significant
Isomer 1 of L-Propargylglycine	1.176300578	0.025881486	0.210164177	Not Significant
Isomer 1 of L-Targinine	0.715882933	0.057892489	0.246326864	Not Significant
Isomer 1 of L-threo-3-Methylaspartic acid	0.884754522	0.475421945	0.535096276	Not Significant
Isomer 1 of L-trans-5-Hydroxy-2-piperidinecarboxylic acid	1.440668524	0.320244752	0.462653305	Not Significant
Isomer 1 of Methionine	1.243744956	0.170796169	0.354235151	Not Significant
Isomer 1 of N5-Acetyl-L-Ornithine	1.151081647	0.535578371	0.566104565	Not Significant
Isomer 1 of Tyrosine	1.001809955	0.979244114	0.703721121	Not Significant
Isomer 2 of Acetylglycine	0.869043959	0.36090121	0.486093193	Not Significant
Isomer 2 of Glutamic Acid	0.997620666	0.968273602	0.702378675	Not Significant
(9Z)-Hexadecenoic Acid	1.086229769	0.558071641	0.574825138	Not Significant
Oleic Acid / Vaccenic Acid	1.126636388	0.717960741	0.624438801	Not Significant
5-Tetradecenoic acid	0.821602252	0.466773656	0.530591322	Not Significant
Isomer 1 of (9Z)-Hexadecenoic Acid	1.164593301	0.630876193	0.592384829	Not Significant
Alanyl-Alanine	1.0272154	0.715123396	0.623729547	Not Significant
Alanyl-Asparagine	0.982037997	0.564099807	0.575693065	Not Significant
Alanyl-Glutamic acid	1.447820343	0.000706818	0.130023696	Not Significant
Alanyl-Glutamine	1.31033123	0.061293118	0.249978299	Not Significant
Alanyl-Histidine	1.150584795	0.380582135	0.49393734	Not Significant
Alanyl-Leucine	1.230030722	0.144751111	0.334756842	Not Significant

Alanyl-Lysine	1.264554164	0.261773228	0.422791013	Not Significant
Alanyl-Proline	1.178780013	0.021895764	0.199455812	Not Significant
Alanyl-Threonine	1.065497495	0.143974468	0.334756842	Not Significant
Asparaginyl-Lysine	0.897652017	0.49748702	0.545776963	Not Significant
Asparaginyl-Tyrosine	1.099659548	0.421953585	0.511740907	Not Significant
Aspartyl-Glycine	1.001363791	0.98431713	0.705276414	Not Significant
Aspartyl-Isoleucine	1.216693419	0.225812259	0.399423482	Not Significant
Glutamyl-Alanine	1.269672131	0.21318515	0.391711917	Not Significant
Glutamyl-Glutamine	0.910059542	0.095659376	0.291137232	Not Significant
Glutamyl-Glycine	1.177401546	0.092309005	0.289048398	Not Significant
Glutamyl-Lysine	1.145348837	0.442278582	0.519434344	Not Significant
Glutamyl-Serine	0.90554943	0.368578678	0.48922427	Not Significant
Glycyl-Arginine	0.894105894	0.11253356	0.306811535	Not Significant
Glycyl-Glutamine	0.835837203	0.033105383	0.221042094	Not Significant
Glycyl-Histidine	0.854950782	0.028538314	0.211124116	Not Significant
Glycyl-Isoleucine	0.973370064	0.639344312	0.59365732	Not Significant
Glycyl-Leucine	0.982148353	0.757242527	0.641114012	Not Significant
Glycyl-Lysine	0.962441315	0.816870905	0.661421591	Not Significant
Glycyl-Phenylalanine	0.931592862	0.441704714	0.519422664	Not Significant
Glycyl-Proline	1.056466997	0.351572031	0.480120394	Not Significant
Glycyl-Threonine	0.983974359	0.913426054	0.688689906	Not Significant
Glycyl-Tryptophan	1.194640863	0.141919124	0.332575052	Not Significant
Glycyl-Tyrosine	0.928835979	0.341392336	0.474885493	Not Significant
Glycyl-Valine	1.045257903	0.629148715	0.592260033	Not Significant
Histidinyl-Arginine	0.9669649	0.568997809	0.577260028	Not Significant
Histidinyl-Aspartate	1.041841004	0.556447755	0.574175763	Not Significant
Histidinyl-Glutamate	1.031972789	0.761065009	0.642611311	Not Significant
Histidinyl-Leucine	1.062808287	0.630470358	0.592260033	Not Significant
Histidinyl-Lysine	1.026427406	0.851083667	0.671511874	Not Significant
Histidinyl-Serine	1.060190476	0.64201447	0.594863963	Not Significant
Histidinyl-Tyrosine	0.826009501	0.291115676	0.447394487	Not Significant
Isoleucyl-Alanine	1.184984782	0.097607244	0.291602876	Not Significant
Isoleucyl-Aspartate	1.116653876	0.222491396	0.398187279	Not Significant
Isoleucyl-Glutamate	0.837997054	0.336879853	0.470417812	Not Significant
Isoleucyl-Glycine	1.0582357	0.662201075	0.602758529	Not Significant
Isoleucyl-Lysine	1.274964838	0.358286706	0.484412555	Not Significant
Isoleucyl-Proline	1.040644945	0.683933201	0.610822228	Not Significant
Isoleucyl-Threonine	0.986550101	0.914021626	0.688689906	Not Significant
Leucyl-Glutamate	1.077873456	0.582491486	0.58222318	Not Significant
Leucyl-Glycine	1.01	0.934947189	0.692990238	Not Significant
Leucyl-Lysine	1.398457584	0.430507433	0.514711366	Not Significant
Leucyl-Proline	1.111823362	0.486059685	0.540765607	Not Significant
Leucyl-Valine	1.226265299	0.395681605	0.50259619	Not Significant
Lysyl-Arginine	1.119402985	0.198589073	0.383371616	Not Significant
Lysyl-Asparagine	0.864760203	0.31217097	0.458373425	Not Significant
Lysyl-Aspartate	0.924899866	0.568419111	0.577260028	Not Significant
Lysyl-Glutamate	0.983056708	0.871168112	0.677452733	Not Significant
Lysyl-Glutamine	0.967305152	0.784697759	0.651499538	Not Significant
Lysyl-Glycine	0.899444989	0.376122303	0.491726898	Not Significant
Lysyl-Isoleucine	1.40903997	0.423918012	0.512032436	Not Significant
Lysyl-Proline	1.132103877	0.462056415	0.530591322	Not Significant

Methionyl-Alanine	1.179698217	0.31624721	0.460265893	Not Significant
Phenylalanyl-Lysine	1.392743222	0.144247643	0.334756842	Not Significant
Prolyl-Alanine	1.14737243	0.227933537	0.401039761	Not Significant
Prolyl-Arginine	0.996003996	0.971901763	0.702378675	Not Significant
Prolyl-Aspartate	0.968023256	0.794917917	0.654651054	Not Significant
Prolyl-Glutamate	1.126546906	0.449082804	0.523736636	Not Significant
Prolyl-Glutamine	1.006772908	0.972682367	0.702378675	Not Significant
Prolyl-Glycine	1.076776165	0.683036365	0.610822228	Not Significant
Prolyl-Lysine	1.118651302	0.42485974	0.512681794	Not Significant
Prolyl-Threonine	0.956455888	0.381394099	0.494399758	Not Significant
Prolyl-Valine	1.089363121	0.336744075	0.470417812	Not Significant
Seryl-Aspartic acid	1.045454545	0.535099718	0.566104565	Not Significant
Seryl-Glycine	1.08660075	0.347649202	0.476261849	Not Significant
Seryl-Histidine	0.983797112	0.912099498	0.688689906	Not Significant
Seryl-Lysine	1.019645609	0.882080746	0.679487121	Not Significant
Seryl-Serine	0.972841901	0.841414727	0.667594134	Not Significant
Seryl-Threonine	0.981651376	0.888734275	0.682432193	Not Significant
Seryl-Tyrosine	0.864599092	0.586854532	0.582825782	Not Significant
Seryl-Valine	1.091497227	0.395250654	0.50259619	Not Significant
Threoninyl-Asparagine	0.978768577	0.778473883	0.649198882	Not Significant
Threoninyl-Histidine	0.762481089	0.124960463	0.318641838	Not Significant
Threoninyl-Isoleucine	1.115825688	0.643731164	0.595691525	Not Significant
Threoninyl-Lysine	0.968111455	0.849109114	0.671184094	Not Significant
Tyrosyl-Alanine	1.10490566	0.240665339	0.409602969	Not Significant
Tyrosyl-Threonine	1.014231775	0.917747196	0.689646319	Not Significant
Tyrosyl-Valine	0.948091248	0.819525519	0.661924282	Not Significant
Valyl-Alanine	1.231590574	0.202472561	0.384788591	Not Significant
Valyl-Aspartate	1.076573161	0.4938358	0.5440351	Not Significant
Valyl-Glutamate	1.014158709	0.903538438	0.686512049	Not Significant
Valyl-Glutamine	0.902055902	0.589529146	0.583066919	Not Significant
Valyl-Glycine	0.952380952	0.672350454	0.606788237	Not Significant
Valyl-Histidine	0.846235419	0.117508197	0.30956656	Not Significant
Valyl-Lysine	1.153128241	0.53038236	0.56463311	Not Significant
Valyl-Valine	1.23538175	0.314058418	0.458697488	Not Significant
prolyl-proline	1.05077187	0.600117903	0.586520137	Not Significant
Isomer 1 of Alanyl-Histidine	1.092710545	0.634790811	0.593381066	Not Significant
Arginyl-Glycine	0.914552737	0.547267815	0.570276153	Not Significant
Asparaginyl-Alanine	2.708180708	0.013177757	0.171457226	Significant Increase
Asparaginyl-Valine	1.34671126	0.13331727	0.328480152	Not Significant
Glutaminyllysine	1.262361624	0.18450278	0.369501475	Not Significant
Glutamylthreonine	0.900027109	0.455161377	0.527364323	Not Significant
Glycyl-Cysteine	1.373452012	0.059939946	0.247999301	Not Significant
Glycyl-Gamma-glutamic acid	1.038284296	0.715986348	0.623729547	Not Significant
Glycyl-glycine	0.781216931	0.055804242	0.24299145	Not Significant
Histidinyl-Glycine	0.86013757	0.2596845	0.421951732	Not Significant
Isoleucyl-Cysteine	1.006975585	0.922583953	0.690347303	Not Significant
L-alpha-Aspartyl-L-hydroxyproline	1.163581286	0.167226478	0.350949185	Not Significant
Prolyl-Asparagine	0.774828512	0.311337155	0.458035	Not Significant
Prolyl-Gamma-glutamic acid	1.06426825	0.640445511	0.593917418	Not Significant
Serylcysteine	0.930101862	0.224130633	0.398187279	Not Significant
Serylproline	0.956262425	0.596278372	0.584501155	Not Significant

Threoninyl-Tryptophan	1.313017306	0.116526072	0.307617488	Not Significant
Tyrosyl-Lysine	0.78389712	0.030223682	0.215741414	Not Significant
Tyrosyl-Serine	0.855731817	0.375290557	0.491726898	Not Significant
gamma-Glutamylglycine	0.92513369	0.435546198	0.518363083	Not Significant
Isomer 1 of Glutamyl-Lysine	1.181406685	0.048697162	0.241458588	Not Significant
Isomer 1 of Glycyl-Lysine	0.91679366	0.406558444	0.506186119	Not Significant
Isomer 1 of Glycyl-Proline	0.766841468	0.248103696	0.414516694	Not Significant
Isomer 1 of Histidinyl-Arginine	0.883183569	0.21126703	0.390427845	Not Significant
Isomer 1 of Isoleucyl-Lysine	0.977521264	0.861955644	0.674276765	Not Significant
Isomer 1 of Phenylalanyl-Lysine	1.119455006	0.64757815	0.59772207	Not Significant
Isomer 1 of Prolyl-Alanine	1.015571591	0.832233723	0.665423608	Not Significant
Isomer 1 of Prolyl-Glycine	0.905003381	0.459716616	0.529222842	Not Significant
Isomer 1 of Prolyl-Valine	1.067256637	0.422900802	0.511834357	Not Significant
Isomer 1 of Threoninyl-Histidine	0.92027027	0.561329131	0.575386502	Not Significant
Acetic Acid	0.772026752	0.089981165	0.285432214	Not Significant
Pyruvic acid	0.812970469	0.010717083	0.166956837	Not Significant
Isomer 1 of Acetic Acid	0.913580247	0.545722121	0.570276153	Not Significant
Alpha-Ketoglutaric Acid	1.273113709	0.055304855	0.24299145	Not Significant
Citric Acid / Isocitric Acid	0.496081277	0.053115803	0.241458588	Not Significant
Fumaric Acid	0.533318274	0.006618049	0.156115145	Significant Decrease
Malic Acid	0.723878518	0.045969439	0.240081416	Not Significant
Succinic Acid	1.440028902	0.175249313	0.358765104	Not Significant
Isomer 1 of Fumaric Acid	0.540578888	0.02595576	0.210164177	Significant Decrease
Isomer 1 of Malic Acid	0.839612859	0.376865459	0.491726898	Not Significant
Pyruvic acid	0.812970469	0.010717083	0.166956837	Not Significant
Isomer 1 of Alpha-Ketoglutaric Acid	1.276153846	0.141631238	0.332575052	Not Significant
Isomer 3 of Alpha-Ketoglutaric Acid	1.186690224	0.162921329	0.346947286	Not Significant
Glyceric Acid	1.153100775	0.323949982	0.464014017	Not Significant
Pyruvic acid	0.812970469	0.010717083	0.166956837	Not Significant
Isomer 1 of Glyceric Acid	0.770577933	0.012123515	0.166956837	Not Significant
Isomer 2 of Glyceric Acid	0.735994633	0.001066123	0.136212565	Not Significant
Alpha-Ketoglutaric Acid	1.273113709	0.055304855	0.24299145	Not Significant
Pyruvic acid	0.812970469	0.010717083	0.166956837	Not Significant
Isomer 1 of Alpha-Ketoglutaric Acid	1.276153846	0.141631238	0.332575052	Not Significant
Isomer 3 of Alpha-Ketoglutaric Acid	1.186690224	0.162921329	0.346947286	Not Significant
Alpha-Ketoglutaric Acid	1.273113709	0.055304855	0.24299145	Not Significant
Threonic Acid	0.973444889	0.846181889	0.669906858	Not Significant
Isomer 1 of Threonic Acid	0.702588802	0.109306605	0.301982658	Not Significant
Pyruvic acid	0.812970469	0.010717083	0.166956837	Not Significant
Isomer 1 of Alpha-Ketoglutaric Acid	1.276153846	0.141631238	0.332575052	Not Significant
Isomer 3 of Alpha-Ketoglutaric Acid	1.186690224	0.162921329	0.346947286	Not Significant
(9Z)-Hexadecenoic Acid	1.086229769	0.558071641	0.574825138	Not Significant
Lauric Acid	0.645661157	0.387252283	0.498084974	Not Significant
Oleic Acid / Vaccenic Acid	1.126636388	0.717960741	0.624438801	Not Significant
Isomer 1 of (9Z)-Hexadecenoic Acid	1.164593301	0.630876193	0.592384829	Not Significant
Oleic Acid / Vaccenic Acid	1.126636388	0.717960741	0.624438801	Not Significant
Acetylglycine	1.128240581	0.561692585	0.575386502	Not Significant
Isomer 1 of Acetylglycine	0.776744186	0.310280503	0.458033123	Not Significant
Isomer 2 of Acetylglycine	0.869043959	0.36090121	0.486093193	Not Significant
3-Hydroxymandelic acid	0.864678899	0.171043769	0.354235151	Not Significant
4-Hydroxybenzoic acid	0.831353231	0.224416148	0.398187279	Not Significant

Tyrosine	1.062776025	0.711045669	0.622572083	Not Significant
3-(4-Hydroxyphenyl)pyruvic acid	0.84588934	0.070529503	0.262519762	Not Significant
3-[(1-Carboxyvinyl)oxy]benzoic acid	0.937741047	0.803024951	0.656703868	Not Significant
N-Acetyl-L-Tyrosine	0.769430725	0.038360449	0.228538487	Not Significant
Isomer 1 of Tyrosine	1.001809955	0.979244114	0.703721121	Not Significant
Alpha-Ketoglutaric Acid	1.273113709	0.055304855	0.24299145	Not Significant
Arginine	1.030175439	0.79888865	0.655879433	Not Significant
Aspartic Acid	0.43049543	0.000376242	0.130023696	Significant Decrease
Citrulline	2.334713187	0.000937354	0.136212565	Significant Increase
Fumaric Acid	0.533318274	0.006618049	0.156115145	Significant Decrease
Glutamic Acid	0.997539543	0.965330284	0.70199957	Not Significant
Isomer 1 of Fumaric Acid	0.540578888	0.02595576	0.210164177	Significant Decrease
Isomer 1 of Glutamic Acid	1.066258552	0.359570677	0.484939943	Not Significant
L-Glutamine / D-Glutamine	1.008454515	0.926636664	0.690759726	Not Significant
L-Ornithine / D-Ornithine	1.598284222	0.061595775	0.249978299	Not Significant
N-Acetyl-L-glutamic acid	1.130388693	0.012539577	0.166956837	Not Significant
N5-Acetyl-L-Ornithine	0.783791866	0.246061144	0.412268944	Not Significant
Isomer 1 of Alpha-Ketoglutaric Acid	1.276153846	0.141631238	0.332575052	Not Significant
Isomer 1 of Arginine	1.092787378	0.362219636	0.486515081	Not Significant
Isomer 1 of L-Ornithine / D-Ornithine	0.716466739	0.032747125	0.219607297	Not Significant
Isomer 1 of N5-Acetyl-L-Ornithine	1.151081647	0.535578371	0.566104565	Not Significant
Isomer 2 of Glutamic Acid	0.997620666	0.968273602	0.702378675	Not Significant
Isomer 3 of Alpha-Ketoglutaric Acid	1.186690224	0.162921329	0.346947286	Not Significant
2'-Deoxyguanosine 5'-monophosphate	0.815384615	0.22199112	0.398187279	Not Significant
Acetylglycine	1.128240581	0.561692585	0.575386502	Not Significant
Hypoxanthine	1.018372703	0.861927576	0.674276765	Not Significant
Xanthine	0.939231738	0.635717707	0.593381066	Not Significant
Adenine	0.846973973	0.35943494	0.484939943	Not Significant
L-Glutamine / D-Glutamine	1.008454515	0.926636664	0.690759726	Not Significant
Oxamic acid	1.002353732	0.972252487	0.702378675	Not Significant
dGDP	0.881795968	0.033637136	0.222357271	Not Significant
Isomer 1 of Acetylglycine	0.776744186	0.310280503	0.458033123	Not Significant
Isomer 1 of Adenine	0.868716931	0.304930735	0.455716044	Not Significant
Isomer 2 of Acetylglycine	0.869043959	0.36090121	0.486093193	Not Significant
Xanthine	0.939231738	0.635717707	0.593381066	Not Significant
Hydracrylic Acid	1.170454545	0.158531659	0.344287374	Not Significant
Uracil	0.787630167	0.075827494	0.270139896	Not Significant
Uridine	0.950813871	0.642390877	0.594958686	Not Significant
Isomer 1 of Uridine	0.892845258	0.423036241	0.511834357	Not Significant
(Z)-3-Peroxyaminoacrylic acid	0.875033378	0.202961215	0.384788591	Not Significant
Aminoacrylic acid	1.442708333	0.313566544	0.458697488	Not Significant
L-Glutamine / D-Glutamine	1.008454515	0.926636664	0.690759726	Not Significant
Orotic acid	0.898434517	0.517365658	0.558271247	Not Significant
Isomer 1 of Aminoacrylic acid	0.765609676	0.032500596	0.219607297	Not Significant
Isomer 2 of Aminoacrylic acid	0.813751669	0.108773248	0.301982658	Not Significant
Alanine	1.201553254	0.002911572	0.148733377	Not Significant
Alpha-Ketoglutaric Acid	1.273113709	0.055304855	0.24299145	Not Significant
Asparagine	0.680398492	0.044774359	0.239384024	Not Significant
Aspartic Acid	0.43049543	0.000376242	0.130023696	Significant Decrease
Citric Acid / Isocitric Acid	0.496081277	0.053115803	0.241458588	Not Significant
Fumaric Acid	0.533318274	0.006618049	0.156115145	Significant Decrease

Gamma-Aminobutyric acid	1.853543307	0.094477943	0.290837295	Not Significant
Glutamic Acid	0.997539543	0.965330284	0.70199957	Not Significant
Succinic Acid	1.440028902	0.175249313	0.358765104	Not Significant
Succinic Semialdehyde	1.412092283	0.176299738	0.359558675	Not Significant
Isomer 1 of Fumaric Acid	0.540578888	0.02595576	0.210164177	Significant Decrease
Isomer 1 of Glutamic Acid	1.066258552	0.359570677	0.484939943	Not Significant
L-Glutamine / D-Glutamine	1.008454515	0.926636664	0.690759726	Not Significant
N-Acetyl-L-aspartic acid	1.551579749	0.155124482	0.342093623	Not Significant
Pyruvic acid	0.812970469	0.010717083	0.166956837	Not Significant
Isomer 1 of Alpha-Ketoglutaric Acid	1.276153846	0.141631238	0.332575052	Not Significant
Isomer 1 of Gamma-Aminobutyric acid	0.941098435	0.755520116	0.641114012	Not Significant
Isomer 2 of Glutamic Acid	0.997620666	0.968273602	0.702378675	Not Significant
Isomer 3 of Alpha-Ketoglutaric Acid	1.186690224	0.162921329	0.346947286	Not Significant
1,3-Diaminopropane	0.490082943	0.026990936	0.211124116	Significant Decrease
5-Aminolevulinic acid	0.846774194	0.081182627	0.274139597	Not Significant
Acetylglycine	1.128240581	0.561692585	0.575386502	Not Significant
Allocystathionine	0.998047511	0.976284617	0.702864616	Not Significant
Alpha-Ketobutyric Acid	0.925006893	0.377013864	0.491726898	Not Significant
Aspartic Acid	0.43049543	0.000376242	0.130023696	Significant Decrease
Choline	0.680555556	0.102936304	0.296827663	Not Significant
Glyceric Acid	1.153100775	0.323949982	0.464014017	Not Significant
Homoserine	0.722604705	0.01955649	0.189453493	Not Significant
Serine	0.802667475	0.094813203	0.290837295	Not Significant
Threonine	0.602526203	0.068006986	0.258168326	Not Significant
Tryptophan	1.031818182	0.852319068	0.671511874	Not Significant
Isomer 1 of Homoserine	0.932373619	0.694047788	0.616741892	Not Significant
5-Hydroxyectoine	1.887267237	0.006986236	0.156115145	Significant Increase
L-2-Amino-3-oxobutanoic acid	0.959542656	0.773502142	0.648570189	Not Significant
L-Aspartate 4-semialdehyde	1.289764359	0.307112715	0.456462048	Not Significant
N(gamma)-Acetyldiaminobutyric acid	1.333333333	0.547675347	0.570276153	Not Significant
N2'-Acetyl-L-Cystathionine	1.078358209	0.554212098	0.572685835	Not Significant
Pyruvic acid	0.812970469	0.010717083	0.166956837	Not Significant
Sarcosine	1.167911775	0.050711515	0.241458588	Not Significant
Isomer 1 of Acetylglycine	0.776744186	0.310280503	0.458033123	Not Significant
Isomer 1 of Glyceric Acid	0.770577933	0.012123515	0.166956837	Not Significant
Isomer 1 of Sarcosine	1.002083333	0.987012956	0.705473687	Not Significant
Isomer 2 of Acetylglycine	0.869043959	0.36090121	0.486093193	Not Significant
Isomer 2 of Glyceric Acid	0.735994633	0.001066123	0.136212565	Not Significant
Isomer 2 of Sarcosine	1.061315496	0.613803607	0.588596602	Not Significant
Arginine	1.030175439	0.79888865	0.655879433	Not Significant
Aspartic Acid	0.43049543	0.000376242	0.130023696	Significant Decrease
Serine	0.802667475	0.094813203	0.290837295	Not Significant
Threonine	0.602526203	0.068006986	0.258168326	Not Significant
Tyrosine	1.062776025	0.711045669	0.622572083	Not Significant
3-(4-Hydroxyphenyl)pyruvic acid	0.84588934	0.070529503	0.262519762	Not Significant
4-Hydroxyphenylglyoxylic acid	0.808349678	0.178216553	0.361429832	Not Significant
L-Aspartate 4-semialdehyde	1.289764359	0.307112715	0.456462048	Not Significant
Pyruvic acid	0.812970469	0.010717083	0.166956837	Not Significant
Isomer 1 of Arginine	1.092787378	0.362219636	0.486515081	Not Significant
Isomer 1 of Tyrosine	1.001809955	0.979244114	0.703721121	Not Significant
5'-Methylthioadenosine	0.747024718	0.257828859	0.420627925	Not Significant

A-Aminobutyric Acid	1.073229292	0.377066156	0.491726898	Not Significant
Alanine	1.201553254	0.002911572	0.148733377	Not Significant
Allocystathionine	0.998047511	0.976284617	0.702864616	Not Significant
Alpha-Ketobutyric Acid	0.925006893	0.377013864	0.491726898	Not Significant
Aspartic Acid	0.43049543	0.000376242	0.130023696	Significant Decrease
Cystine	0.878081279	0.47193769	0.532933308	Not Significant
Glutathione	0.633864947	0.057203595	0.24508997	Not Significant
Homoserine	0.722604705	0.01955649	0.189453493	Not Significant
Methionine	0.870646766	0.145357826	0.334756842	Not Significant
Methionine Sulfoxide	1.154382105	0.321917919	0.4627078	Not Significant
S-Adenosylhomocysteine	0.804871591	0.345252384	0.475687508	Not Significant
Serine	0.802667475	0.094813203	0.290837295	Not Significant
Isomer 1 of Homoserine	0.932373619	0.694047788	0.616741892	Not Significant
1,2-Dihydroxy-5-(methylthio)pent-1-en-3-one	0.848891874	0.383978097	0.495676663	Not Significant
1-Aminocyclopropane-1-carboxylic acid	0.705864929	0.139494568	0.331131602	Not Significant
3-Sulfino-L-alanine	0.838729571	0.058927179	0.24795085	Not Significant
Dehydroalanine	0.857874957	0.175809273	0.358926788	Not Significant
L-Aspartate 4-semialdehyde	1.289764359	0.307112715	0.456462048	Not Significant
L-Cysteic acid	0.926796953	0.17716072	0.360636737	Not Significant
L-Homocysteine	0.923754659	0.677968182	0.609188801	Not Significant
N-Acetyl-Dehydroalanine	1.151295337	0.168562882	0.351486087	Not Significant
N-Acetyl-L-Methionine	0.753605769	0.028543868	0.211124116	Not Significant
N2'-Acetyl-L-Cystathionine	1.078358209	0.554212098	0.572685835	Not Significant
O-Acetyl-L-homoserine	0.780012571	0.115208587	0.307279086	Not Significant
Ophthalmic acid	1.299809524	0.241122442	0.410059325	Not Significant
Pyruvic acid	0.812970469	0.010717083	0.166956837	Not Significant
S-Glutathionyl-L-cysteine - 2 tags / Cysteineglutathione disulfide	1.321806347	0.109381737	0.301982658	Not Significant
Isomer 1 of Glutathione	0.892248311	0.198418749	0.383371616	Not Significant
Isomer 1 of Methionine	1.243744956	0.170796169	0.354235151	Not Significant
Alloisoleucine	0.938195303	0.653398811	0.598764957	Not Significant
Leucine	0.977616646	0.822884278	0.663572912	Not Significant
Valine	1.079760718	0.232964721	0.403460817	Not Significant
2-Hydroxy-2-Methylbutanedioic Acid	0.911891892	0.211946544	0.390427845	Not Significant
2-Isopropylmalic Acid	1.763522013	0.088997103	0.284423732	Not Significant
Alloisoleucine	0.938195303	0.653398811	0.598764957	Not Significant
Alpha-Ketobutyric Acid	0.925006893	0.377013864	0.491726898	Not Significant
Leucine	0.977616646	0.822884278	0.663572912	Not Significant
Mesaconic Acid	1.280523256	0.139624616	0.331131602	Not Significant
Threonine	0.602526203	0.068006986	0.258168326	Not Significant
Valine	1.079760718	0.232964721	0.403460817	Not Significant
Pyruvic acid	0.812970469	0.010717083	0.166956837	Not Significant
Alpha-Ketoglutaric Acid	1.273113709	0.055304855	0.24299145	Not Significant
Aspartic Acid	0.43049543	0.000376242	0.130023696	Significant Decrease
Homoserine	0.722604705	0.01955649	0.189453493	Not Significant
Lysine	1.251472116	0.043063596	0.236691698	Not Significant
Saccharopine	2.554776512	0.040139537	0.232895172	Significant Increase
Isomer 1 of Homoserine	0.932373619	0.694047788	0.616741892	Not Significant
L-2-Amino-6-oxoheptanedioic acid	1.446071254	0.288504805	0.446215008	Not Significant
L-2-Aminoadipate 6-semialdehyde / Allysine	1.155949367	0.04554703	0.240081416	Not Significant
L-Aspartate 4-semialdehyde	1.289764359	0.307112715	0.456462048	Not Significant
Isomer 1 of Alpha-Ketoglutaric Acid	1.276153846	0.141631238	0.332575052	Not Significant

Isomer 3 of Alpha-Ketoglutaric Acid	1.186690224	0.162921329	0.346947286	Not Significant
5-Aminopentanoic acid	1.156578947	0.284539903	0.442935144	Not Significant
Acetylglycine	1.128240581	0.561692585	0.575386502	Not Significant
Alpha-Ketoglutaric Acid	1.273113709	0.055304855	0.24299145	Not Significant
Lysine	1.251472116	0.043063596	0.236691698	Not Significant
N6-Acetyl-Lysine	1.256925996	0.037968822	0.228538487	Not Significant
Pipecollinic Acid	1.205513784	0.056957161	0.244746612	Not Significant
Saccharopine	2.554776512	0.040139537	0.232895172	Significant Increase
Succinic Acid	1.440028902	0.175249313	0.358765104	Not Significant
Isomer 1 of Pipecollinic Acid	1.092413236	0.501769374	0.548810252	Not Significant
(3S,5S)-3,5-Diaminohexanoic acid	1.319668737	0.068893608	0.258906388	Not Significant
(S)-2,3,4,5-Tetrahydropyridine-2-carboxylic acid	1.088671612	0.33211234	0.468490149	Not Significant
(S)-5-Amino-3-oxohexanoic acid	1.264720435	0.428369425	0.514392927	Not Significant
2-Amino-5-oxohexanoic acid	1.119609439	0.504843124	0.550230828	Not Significant
5-Aminopentanal	1.113561191	0.157384229	0.343585288	Not Significant
5-Aminopentanamide	0.978568064	0.787025065	0.651997654	Not Significant
6-Amino-2-oxohexanoic acid	1.037185563	0.660588046	0.601795155	Not Significant
D-Lysopine	1.856756757	0.001396603	0.136212565	Significant Increase
Delta1-Piperideine-2-carboxylic acid	1.044580153	0.813691685	0.660112518	Not Significant
L-2-Aminoadipate 6-semialdehyde / Allysine	1.155949367	0.04554703	0.240081416	Not Significant
Ne,Ne dimethyllysine	0.959824047	0.795481375	0.654651054	Not Significant
Isomer 1 of (S)-2,3,4,5-Tetrahydropyridine-2-carboxylic acid	1.148992402	0.166883176	0.350567756	Not Significant
Isomer 1 of 5-Aminopentanal	1.026910657	0.147138114	0.335741017	Not Significant
Isomer 1 of 5-Aminopentanamide	1.090432099	0.294079786	0.449403617	Not Significant
Isomer 1 of Acetylglycine	0.776744186	0.310280503	0.458033123	Not Significant
Isomer 1 of Alpha-Ketoglutaric Acid	1.276153846	0.141631238	0.332575052	Not Significant
Isomer 1 of Delta1-Piperideine-2-carboxylic acid	1.10833079	0.002716502	0.148733377	Not Significant
Isomer 2 of (S)-2,3,4,5-Tetrahydropyridine-2-carboxylic acid	1.202155333	0.03034741	0.215914363	Not Significant
Isomer 2 of Acetylglycine	0.869043959	0.36090121	0.486093193	Not Significant
Isomer 3 of Alpha-Ketoglutaric Acid	1.186690224	0.162921329	0.346947286	Not Significant
1,3-Diaminopropane	0.490082943	0.026990936	0.211124116	Significant Decrease
4-Hydroxyproline	1.024486226	0.921623083	0.690347303	Not Significant
5-Aminopentanoic acid	1.156578947	0.284539903	0.442935144	Not Significant
Arginine	1.030175439	0.79888865	0.655879433	Not Significant
Gamma-Aminobutyric acid	1.853543307	0.094477943	0.290837295	Not Significant
Glutamic Acid	0.997539543	0.965330284	0.70199957	Not Significant
N-Acetylputrescine	0.605621806	0.043514923	0.236887617	Significant Decrease
Proline	0.958639093	0.417102067	0.510778308	Not Significant
Isomer 1 of Glutamic Acid	1.066258552	0.359570677	0.484939943	Not Significant
4-Aminobutyraldehyde	0.8944	0.666778191	0.604809887	Not Significant
4-Guanidinobutanal	0.972885033	0.654562031	0.599324729	Not Significant
4-Oxoproline	0.983196159	0.611519349	0.58794727	Not Significant
5-Amino-2-oxopentanoic acid	1.0784	0.781192394	0.650011248	Not Significant
Feruloylputrescine	0.854545455	0.19526326	0.381683741	Not Significant
L-1-Pyrroline-3-hydroxy-5-carboxylic acid	0.898876404	0.464902782	0.530591322	Not Significant
L-Aspartate 4-semialdehyde	1.289764359	0.307112715	0.456462048	Not Significant
L-Ornithine / D-Ornithine	1.598284222	0.061595775	0.249978299	Not Significant
L-erythro-4-Hydroxyglutamic acid / 4-Hydroxy-L-glutamic acid	0.615539568	0.038011344	0.228538487	Significant Decrease

N-Acetyl-2-Oxo-4-hydroxy-5-aminovaleric acid	1.177274669	0.328791788	0.466021019	Not Significant
N5-Acetyl-L-Ornithine	0.783791866	0.246061144	0.412268944	Not Significant
Pyruvic acid	0.812970469	0.010717083	0.166956837	Not Significant
Sarcosine	1.167911775	0.050711515	0.241458588	Not Significant
Spermidine	0.342320966	0.065268951	0.252446023	Not Significant
p-Coumaroylputrescine	1.340101523	0.192964865	0.378259943	Not Significant
Isomer 1 of 4-Guanidinobutanal	1.897267206	0.012431817	0.166956837	Significant Increase
Isomer 1 of Arginine	1.092787378	0.362219636	0.486515081	Not Significant
Isomer 1 of Gamma-Aminobutyric acid	0.941098435	0.755520116	0.641114012	Not Significant
Isomer 1 of L-1-Pyrroline-3-hydroxy-5-carboxylic acid	1.165991903	0.325208006	0.464014017	Not Significant
Isomer 1 of L-Ornithine / D-Ornithine	0.716466739	0.032747125	0.219607297	Not Significant
Isomer 1 of N-Acetylputrescine	0.997278912	0.972503674	0.702378675	Not Significant
Isomer 1 of N5-Acetyl-L-Ornithine	1.151081647	0.535578371	0.566104565	Not Significant
Isomer 1 of Sarcosine	1.002083333	0.987012956	0.705473687	Not Significant
Isomer 2 of Glutamic Acid	0.997620666	0.968273602	0.702378675	Not Significant
Isomer 2 of Sarcosine	1.061315496	0.613803607	0.588596602	Not Significant
Glutamic Acid	0.997539543	0.965330284	0.70199957	Not Significant
Proline	0.958639093	0.417102067	0.510778308	Not Significant
Isomer 1 of Glutamic Acid	1.066258552	0.359570677	0.484939943	Not Significant
Isomer 2 of Glutamic Acid	0.997620666	0.968273602	0.702378675	Not Significant
Alpha-Ketoglutaric Acid	1.273113709	0.055304855	0.24299145	Not Significant
Aspartic Acid	0.43049543	0.000376242	0.130023696	Significant Decrease
Glutamic Acid	0.997539543	0.965330284	0.70199957	Not Significant
Histidine	0.934123847	0.486890682	0.541266793	Not Significant
Imidazolepyruvic Acid	0.741756719	0.098983496	0.292664763	Not Significant
Isomer 1 of Glutamic Acid	1.066258552	0.359570677	0.484939943	Not Significant
Dihydrourocanic acid	2.35942029	0.011506466	0.166956837	Significant Increase
Histamine	2.589236683	0.006183235	0.156115145	Significant Increase
N-Formimino-L-glutamic acid	1.404237979	0.038975946	0.230457229	Not Significant
N-Formyl-L-aspartic acid	1.174224344	0.112114266	0.306408006	Not Significant
beta-Alanyl-N(pi)-methyl-L-histidine	0.961895613	0.783670806	0.651058824	Not Significant
Isomer 1 of Alpha-Ketoglutaric Acid	1.276153846	0.141631238	0.332575052	Not Significant
Isomer 1 of Histidine	0.996963842	0.970509713	0.702378675	Not Significant
Isomer 2 of Glutamic Acid	0.997620666	0.968273602	0.702378675	Not Significant
Isomer 3 of Alpha-Ketoglutaric Acid	1.186690224	0.162921329	0.346947286	Not Significant
3-Hydroxymandelic acid	0.864678899	0.171043769	0.354235151	Not Significant
3-Hydroxyphenylacetic acid	0.85	0.257237486	0.420627925	Not Significant
Fumaric Acid	0.533318274	0.006618049	0.156115145	Significant Decrease
Phenol	0.910515215	0.610918579	0.587630016	Not Significant
Succinic Acid	1.440028902	0.175249313	0.358765104	Not Significant
Succinic Semialdehyde	1.412092283	0.176299738	0.359558675	Not Significant
Tyrosine	1.062776025	0.711045669	0.622572083	Not Significant
Isomer 1 of Fumaric Acid	0.540578888	0.02595576	0.210164177	Significant Decrease
3,4-Dihydroxymandelaldehyde	0.714451202	0.10468059	0.297648105	Not Significant
3-(4-Hydroxyphenyl)pyruvic acid	0.84588934	0.070529503	0.262519762	Not Significant
4-Hydroxyphenylethanol	0.736061381	0.15589684	0.34344786	Not Significant
Hydroquinone	1.370600414	0.332077587	0.468490149	Not Significant
N-Acetyl-Dopamine	0.993842709	0.944658141	0.694854798	Not Significant
N-Acetyl-L-Tyrosine	0.769430725	0.038360449	0.228538487	Not Significant
Pyruvic acid	0.812970469	0.010717083	0.166956837	Not Significant

Isomer 1 of Tyrosine	1.001809955	0.979244114	0.703721121	Not Significant
3-Hydroxyphenylacetic acid	0.85	0.257237486	0.420627925	Not Significant
4-Hydroxybenzoic acid	0.831353231	0.224416148	0.398187279	Not Significant
Fumaric Acid	0.533318274	0.006618049	0.156115145	Significant Decrease
Phenylalanine	0.914249283	0.436039882	0.518363083	Not Significant
Phenylpyruvic Acid	1.049965302	0.769906342	0.646804786	Not Significant
Salicylic Acid	1.171747967	0.244864356	0.412198411	Not Significant
Succinic Acid	1.440028902	0.175249313	0.358765104	Not Significant
Tyrosine	1.062776025	0.711045669	0.622572083	Not Significant
Isomer 1 of Fumaric Acid	0.540578888	0.02595576	0.210164177	Significant Decrease
2-Hydroxy-2,4-pentadienoic acid	0.926312006	0.534032374	0.566104565	Not Significant
8-Methyl-6-nonenoic acid	1.226905515	0.104222175	0.297610096	Not Significant
N-Acetyl-L-Tyrosine	0.769430725	0.038360449	0.228538487	Not Significant
Phenylacetylglycine	1.029221882	0.811046256	0.659124848	Not Significant
Pyruvic acid	0.812970469	0.010717083	0.166956837	Not Significant
Isomer 1 of Tyrosine	1.001809955	0.979244114	0.703721121	Not Significant
Glycolic Acid	1.137090909	0.406084342	0.506186119	Not Significant
Phenol	0.910515215	0.610918579	0.587630016	Not Significant
Succinic Acid	1.440028902	0.175249313	0.358765104	Not Significant
Hydroquinone	1.370600414	0.332077587	0.468490149	Not Significant
Isomer 1 of Glycolic Acid	0.981711097	0.803915945	0.656703868	Not Significant
4-Hydroxybenzoic acid	0.831353231	0.224416148	0.398187279	Not Significant
Cis,Cis-Muconic Acid	0.835214447	0.306009479	0.45638527	Not Significant
Phenol	0.910515215	0.610918579	0.587630016	Not Significant
Protocatechuic acid	1.351843625	0.353682316	0.482092101	Not Significant
(S)-5-Oxo-2,5-dihydrofuran-2-acetic acid	1.398496241	0.046822045	0.240081416	Not Significant
2-Hydroxy-2,4-pentadienoic acid	0.926312006	0.534032374	0.566104565	Not Significant
Hydroquinone	1.370600414	0.332077587	0.468490149	Not Significant
Pyruvic acid	0.812970469	0.010717083	0.166956837	Not Significant
3-Fluorocatechol	0.609913793	0.016476365	0.179915919	Significant Decrease
2-Aminobenzoic acid	0.594040968	0.005676502	0.156115145	Significant Decrease
3-Hydroxyanthranilic acid	0.654390935	0.062383406	0.249978299	Not Significant
Kynurenine	0.766063631	0.238040758	0.406731059	Not Significant
Picolinic Acid	1.167371388	0.008749802	0.162966298	Not Significant
Tryptophan	1.031818182	0.852319068	0.671511874	Not Significant
2-Aminomuconic acid	1.347650514	0.14044553	0.332154035	Not Significant
5-Hydroxyindoleacetic acid	1.048702453	0.798745598	0.655879433	Not Significant
5-Hydroxykynurenine	1.468461822	0.138657011	0.330785934	Not Significant
7,8-Dihydroxykynurenic acid	0.836311423	0.027420217	0.211124116	Not Significant
Formyl-5-hydroxykynurenamine	0.712606303	0.091977451	0.289048398	Not Significant
N-Acetyl-2-Aminomuconate semialdehyde	1.074231524	0.593491588	0.583066919	Not Significant
N-Acetylindoxyl	1.224442988	0.45002778	0.524019407	Not Significant
Isomer 1 of 3-Hydroxyanthranilic acid	0.966863034	0.621937823	0.591149454	Not Significant
Isomer 1 of 5-Hydroxyindoleacetic acid	0.865030675	0.550339578	0.571357952	Not Significant
2-Aminobenzoic acid	0.594040968	0.005676502	0.156115145	Significant Decrease
Phenylalanine	0.914249283	0.436039882	0.518363083	Not Significant
Phenylpyruvic Acid	1.049965302	0.769906342	0.646804786	Not Significant
Protocatechuic acid	1.351843625	0.353682316	0.482092101	Not Significant
Tryptophan	1.031818182	0.852319068	0.671511874	Not Significant
Tyrosine	1.062776025	0.711045669	0.622572083	Not Significant
3-(4-Hydroxyphenyl)pyruvic acid	0.84588934	0.070529503	0.262519762	Not Significant

L-Aspartate 4-semialdehyde	1.289764359	0.307112715	0.456462048	Not Significant
N-Acetyl-L-Tyrosine	0.769430725	0.038360449	0.228538487	Not Significant
Isomer 1 of Tyrosine	1.001809955	0.979244114	0.703721121	Not Significant
Proline	0.958639093	0.417102067	0.510778308	Not Significant
Tyrosine	1.062776025	0.711045669	0.622572083	Not Significant
3-(4-Hydroxyphenyl)pyruvic acid	0.84588934	0.070529503	0.262519762	Not Significant
3-Methylpyrrole-2,4-dicarboxylic acid	1.053668478	0.421649679	0.511740907	Not Significant
Isomer 1 of 3-Methylpyrrole-2,4-dicarboxylic acid	0.993579945	0.95139995	0.69653775	Not Significant
Isomer 1 of Tyrosine	1.001809955	0.979244114	0.703721121	Not Significant
1,3-Diaminopropane	0.490082943	0.026990936	0.211124116	Significant Decrease
Aspartic Acid	0.43049543	0.000376242	0.130023696	Significant Decrease
Gamma-Aminobutyric acid	1.853543307	0.094477943	0.290837295	Not Significant
Histidine	0.934123847	0.486890682	0.541266793	Not Significant
Hydracrylic Acid	1.170454545	0.158531659	0.344287374	Not Significant
Uracil	0.787630167	0.075827494	0.270139896	Not Significant
4-Aminobutyraldehyde	0.8944	0.666778191	0.604809887	Not Significant
Spermidine	0.342320966	0.065268951	0.252446023	Not Significant
beta-Alanyl-N(pi)-methyl-L-histidine	0.961895613	0.783670806	0.651058824	Not Significant
Isomer 1 of Gamma-Aminobutyric acid	0.941098435	0.755520116	0.641114012	Not Significant
Isomer 1 of Histidine	0.996963842	0.970509713	0.702378675	Not Significant
Acetic Acid	0.772026752	0.089981165	0.285432214	Not Significant
Alanine	1.201553254	0.002911572	0.148733377	Not Significant
Alpha-Ketoglutaric Acid	1.273113709	0.055304855	0.24299145	Not Significant
Glutamic Acid	0.997539543	0.965330284	0.70199957	Not Significant
Isomer 1 of Glutamic Acid	1.066258552	0.359570677	0.484939943	Not Significant
3-Sulfino-L-alanine	0.838729571	0.058927179	0.24795085	Not Significant
L-Cysteic acid	0.926796953	0.17716072	0.360636737	Not Significant
Pyruvic acid	0.812970469	0.010717083	0.166956837	Not Significant
Isomer 1 of Acetic Acid	0.913580247	0.545722121	0.570276153	Not Significant
Isomer 1 of Alpha-Ketoglutaric Acid	1.276153846	0.141631238	0.332575052	Not Significant
Isomer 2 of Glutamic Acid	0.997620666	0.968273602	0.702378675	Not Significant
Isomer 3 of Alpha-Ketoglutaric Acid	1.186690224	0.162921329	0.346947286	Not Significant
Acetic Acid	0.772026752	0.089981165	0.285432214	Not Significant
Acetylglycine	1.128240581	0.561692585	0.575386502	Not Significant
3-Phosphonopyruvic acid	1.170272436	0.101473804	0.295965261	Not Significant
Pyruvic acid	0.812970469	0.010717083	0.166956837	Not Significant
Isomer 1 of Acetic Acid	0.913580247	0.545722121	0.570276153	Not Significant
Isomer 1 of Acetylglycine	0.776744186	0.310280503	0.458033123	Not Significant
Isomer 2 of Acetylglycine	0.869043959	0.36090121	0.486093193	Not Significant
Alanine	1.201553254	0.002911572	0.148733377	Not Significant
Alloisoleucine	0.938195303	0.653398811	0.598764957	Not Significant
Asparagine	0.680398492	0.044774359	0.239384024	Not Significant
Aspartic Acid	0.43049543	0.000376242	0.130023696	Significant Decrease
Glutamic Acid	0.997539543	0.965330284	0.70199957	Not Significant
Phenylalanine	0.914249283	0.436039882	0.518363083	Not Significant
Serine	0.802667475	0.094813203	0.290837295	Not Significant
Tyrosine	1.062776025	0.711045669	0.622572083	Not Significant
Valine	1.079760718	0.232964721	0.403460817	Not Significant
Isomer 1 of Glutamic Acid	1.066258552	0.359570677	0.484939943	Not Significant
3-Cyano-L-alanine	0.725131701	0.026974526	0.211124116	Not Significant
N-Hydroxy-L-phenylalanine	1.082248829	0.601570444	0.586520137	Not Significant

gamma-Amino-gamma-cyanobutanoic acid	0.957599713	0.616136899	0.588789334	Not Significant
Isomer 1 of 3-Cyano-L-alanine	0.921280854	0.291328747	0.447405082	Not Significant
Isomer 1 of Tyrosine	1.001809955	0.979244114	0.703721121	Not Significant
Isomer 2 of Glutamic Acid	0.997620666	0.968273602	0.702378675	Not Significant
Alpha-Ketoglutaric Acid	1.273113709	0.055304855	0.24299145	Not Significant
Glutamic Acid	0.997539543	0.965330284	0.70199957	Not Significant
Pyroglutamic Acid	0.816136773	0.055737599	0.24299145	Not Significant
Isomer 1 of Glutamic Acid	1.066258552	0.359570677	0.484939943	Not Significant
L-Glutamine / D-Glutamine	1.008454515	0.926636664	0.690759726	Not Significant
Isomer 1 of Alpha-Ketoglutaric Acid	1.276153846	0.141631238	0.332575052	Not Significant
Isomer 2 of Glutamic Acid	0.997620666	0.968273602	0.702378675	Not Significant
Isomer 3 of Alpha-Ketoglutaric Acid	1.186690224	0.162921329	0.346947286	Not Significant
Arginine	1.030175439	0.79888865	0.655879433	Not Significant
5-Amino-2-oxopentanoic acid	1.0784	0.781192394	0.650011248	Not Significant
L-Ornithine / D-Ornithine	1.598284222	0.061595775	0.249978299	Not Significant
N5-Acetyl-L-Ornithine	0.783791866	0.246061144	0.412268944	Not Significant
Isomer 1 of Arginine	1.092787378	0.362219636	0.486515081	Not Significant
Isomer 1 of L-Ornithine / D-Ornithine	0.716466739	0.032747125	0.219607297	Not Significant
Isomer 1 of N5-Acetyl-L-Ornithine	1.151081647	0.535578371	0.566104565	Not Significant
Alanine	1.201553254	0.002911572	0.148733377	Not Significant
Alanyl-Alanine	1.0272154	0.715123396	0.623729547	Not Significant
Pyruvic acid	0.812970469	0.010717083	0.166956837	Not Significant
Acetylglycine	1.128240581	0.561692585	0.575386502	Not Significant
Glutamic Acid	0.997539543	0.965330284	0.70199957	Not Significant
Glutathione	0.633864947	0.057203595	0.24508997	Not Significant
Oxidized glutathione	0.98633678	0.830115767	0.665423608	Not Significant
Pyroglutamic Acid	0.816136773	0.055737599	0.24299145	Not Significant
Isomer 1 of Glutamic Acid	1.066258552	0.359570677	0.484939943	Not Significant
Bis-gamma-glutamylcystine	1.260854817	0.252797745	0.417799777	Not Significant
L-Ornithine / D-Ornithine	1.598284222	0.061595775	0.249978299	Not Significant
N5-Acetyl-L-Ornithine	0.783791866	0.246061144	0.412268944	Not Significant
Spermidine	0.342320966	0.065268951	0.252446023	Not Significant
Isomer 1 of Acetylglycine	0.776744186	0.310280503	0.458033123	Not Significant
Isomer 1 of Glutathione	0.892248311	0.198418749	0.383371616	Not Significant
Isomer 1 of L-Ornithine / D-Ornithine	0.716466739	0.032747125	0.219607297	Not Significant
Isomer 1 of N5-Acetyl-L-Ornithine	1.151081647	0.535578371	0.566104565	Not Significant
Isomer 2 of Acetylglycine	0.869043959	0.36090121	0.486093193	Not Significant
Isomer 2 of Glutamic Acid	0.997620666	0.968273602	0.702378675	Not Significant
Glutamic Acid	0.997539543	0.965330284	0.70199957	Not Significant
Isomer 1 of Glutamic Acid	1.066258552	0.359570677	0.484939943	Not Significant
2-Deoxy-scyllo-inosamine	1.106274937	0.310553664	0.458035	Not Significant
4'-Oxonebramine	1.005778259	0.96186033	0.70065019	Not Significant
Isomer 2 of Glutamic Acid	0.997620666	0.968273602	0.702378675	Not Significant
Alanine	1.201553254	0.002911572	0.148733377	Not Significant
Alanyl-Alanine	1.0272154	0.715123396	0.623729547	Not Significant
Glyceric Acid	1.153100775	0.323949982	0.464014017	Not Significant
Isomer 1 of Glyceric Acid	0.770577933	0.012123515	0.166956837	Not Significant
Isomer 2 of Glyceric Acid	0.735994633	0.001066123	0.136212565	Not Significant
Choline	0.680555556	0.102936304	0.296827663	Not Significant
Ethanolamine	0.848249027	0.3093469	0.457715956	Not Significant
Diethanolamine	0.9606249	0.945257146	0.694854798	Not Significant

9-Oxononanoic acid	0.958357102	0.803687315	0.656703868	Not Significant
Traumatic acid	0.849753695	0.509539803	0.554049501	Not Significant
Traumatin	1.40745098	0.211663631	0.390427845	Not Significant
Isomer 1 of Traumatin	0.890768147	0.65958157	0.601383196	Not Significant
Serine	0.802667475	0.094813203	0.290837295	Not Significant
2-Isopropylmalic Acid	1.763522013	0.088997103	0.284423732	Not Significant
Acetic Acid	0.772026752	0.089981165	0.285432214	Not Significant
Fumaric Acid	0.533318274	0.006618049	0.156115145	Significant Decrease
Malic Acid	0.723878518	0.045969439	0.240081416	Not Significant
Succinic Acid	1.440028902	0.175249313	0.358765104	Not Significant
Isomer 1 of Fumaric Acid	0.540578888	0.02595576	0.210164177	Significant Decrease
Isomer 1 of Malic Acid	0.839612859	0.376865459	0.491726898	Not Significant
Pyruvic acid	0.812970469	0.010717083	0.166956837	Not Significant
Isomer 1 of Acetic Acid	0.913580247	0.545722121	0.570276153	Not Significant
2-Aminobenzoic acid	0.594040968	0.005676502	0.156115145	Significant Decrease
Salicylic Acid	1.171747967	0.244864356	0.412198411	Not Significant
2'-Aminobiphenyl-2,3-diol	0.492043759	0.008121119	0.160207537	Significant Decrease
2-Hydroxy-2,4-pentadienoic acid	0.926312006	0.534032374	0.566104565	Not Significant
Pyruvic acid	0.812970469	0.010717083	0.166956837	Not Significant
2-Hydroxy-2,4-pentadienoic acid	0.926312006	0.534032374	0.566104565	Not Significant
Pyruvic acid	0.812970469	0.010717083	0.166956837	Not Significant
4-Hydroxybenzoic acid	0.831353231	0.224416148	0.398187279	Not Significant
Protocatechuic acid	1.351843625	0.353682316	0.482092101	Not Significant
o-Cresol	0.889884393	0.528504174	0.563287848	Not Significant
4-Hydroxy-3-methylbenzaldehyde	1.027653411	0.837992213	0.667393279	Not Significant
Glycolic Acid	1.137090909	0.406084342	0.506186119	Not Significant
Isomer 1 of Glycolic Acid	0.981711097	0.803915945	0.656703868	Not Significant
Salicylic Acid	1.171747967	0.244864356	0.412198411	Not Significant
1,2-Dihydroxynaphthalene-6-sulfonic acid	1.169892473	0.22947079	0.401897993	Not Significant
4-Hydroxymethylcatechol	0.812140115	0.051877364	0.241458588	Not Significant
cis-2-Carboxycyclohexyl-acetic acid	1.384581691	0.012312717	0.166956837	Not Significant
Isomer 1 of cis-2-Carboxycyclohexyl-acetic acid	1.290529154	0.105080342	0.29768191	Not Significant
2-Aminobenzoic acid	0.594040968	0.005676502	0.156115145	Significant Decrease
3-Hydroxyanthranilic acid	0.654390935	0.062383406	0.249978299	Not Significant
4-Hydroxybenzoic acid	0.831353231	0.224416148	0.398187279	Not Significant
Guaiacol	0.827110663	0.553942565	0.572680022	Not Significant
Phenol	0.910515215	0.610918579	0.587630016	Not Significant
Protocatechuic acid	1.351843625	0.353682316	0.482092101	Not Significant
Terephthalic Acid	1.250477403	0.039669723	0.231813188	Not Significant
Vanillic acid	0.994049306	0.969402049	0.702378675	Not Significant
4-Aminobenzoic acid	0.909681611	0.158744435	0.344287374	Not Significant
4-Hydroxyphenylglyoxylic acid	0.808349678	0.178216553	0.361429832	Not Significant
Hydroquinone	1.370600414	0.332077587	0.468490149	Not Significant
Isomer 1 of 3-Hydroxyanthranilic acid	0.966863034	0.621937823	0.591149454	Not Significant
Acetic Acid	0.772026752	0.089981165	0.285432214	Not Significant
Acetylglycine	1.128240581	0.561692585	0.575386502	Not Significant
Alpha-Ketoglutaric Acid	1.273113709	0.055304855	0.24299145	Not Significant
Citric Acid / Isocitric Acid	0.496081277	0.053115803	0.241458588	Not Significant
Glutamic Acid	0.997539543	0.965330284	0.70199957	Not Significant
Glyceric Acid	1.153100775	0.323949982	0.464014017	Not Significant
Glycolic Acid	1.137090909	0.406084342	0.506186119	Not Significant

Malic Acid	0.723878518	0.045969439	0.240081416	Not Significant
Mesaconic Acid	1.280523256	0.139624616	0.331131602	Not Significant
Serine	0.802667475	0.094813203	0.290837295	Not Significant
Succinic Acid	1.440028902	0.175249313	0.358765104	Not Significant
Isomer 1 of Glutamic Acid	1.066258552	0.359570677	0.484939943	Not Significant
Isomer 1 of Malic Acid	0.839612859	0.376865459	0.491726898	Not Significant
L-Glutamine / D-Glutamine	1.008454515	0.926636664	0.690759726	Not Significant
L-threo-3-Methylaspartic acid	0.905589343	0.102236778	0.296827663	Not Significant
Pyruvic acid	0.812970469	0.010717083	0.166956837	Not Significant
Isomer 1 of Acetic Acid	0.913580247	0.545722121	0.570276153	Not Significant
Isomer 1 of Acetylglycine	0.776744186	0.310280503	0.458033123	Not Significant
Isomer 1 of Alpha-Ketoglutaric Acid	1.276153846	0.141631238	0.332575052	Not Significant
Isomer 1 of Glyceric Acid	0.770577933	0.012123515	0.166956837	Not Significant
Isomer 1 of Glycolic Acid	0.981711097	0.803915945	0.656703868	Not Significant
Isomer 1 of L-threo-3-Methylaspartic acid	0.884754522	0.475421945	0.535096276	Not Significant
Isomer 2 of Acetylglycine	0.869043959	0.36090121	0.486093193	Not Significant
Isomer 2 of Glutamic Acid	0.997620666	0.968273602	0.702378675	Not Significant
Isomer 2 of Glyceric Acid	0.735994633	0.001066123	0.136212565	Not Significant
Isomer 3 of Alpha-Ketoglutaric Acid	1.186690224	0.162921329	0.346947286	Not Significant
2,4-Diamino-6-hydroxylaminotoluene	0.643929059	0.013619016	0.171457226	Significant Decrease
2,4-Diaminotoluene	1.079506511	0.678504499	0.609232429	Not Significant
Acetic Acid	0.772026752	0.089981165	0.285432214	Not Significant
Alpha-Ketobutyric Acid	0.925006893	0.377013864	0.491726898	Not Significant
Hydracrylic Acid	1.170454545	0.158531659	0.344287374	Not Significant
Succinic Acid	1.440028902	0.175249313	0.358765104	Not Significant
Isomer 1 of Acetic Acid	0.913580247	0.545722121	0.570276153	Not Significant
Alpha-Ketoglutaric Acid	1.273113709	0.055304855	0.24299145	Not Significant
Butanoic Acid / Isobutyric Acid	1.007497657	0.939568445	0.69345528	Not Significant
Fumaric Acid	0.533318274	0.006618049	0.156115145	Significant Decrease
Gamma-Aminobutyric acid	1.853543307	0.094477943	0.290837295	Not Significant
Glutamic Acid	0.997539543	0.965330284	0.70199957	Not Significant
Malic Acid	0.723878518	0.045969439	0.240081416	Not Significant
Succinic Acid	1.440028902	0.175249313	0.358765104	Not Significant
Succinic Semialdehyde	1.412092283	0.176299738	0.359558675	Not Significant
Isomer 1 of Fumaric Acid	0.540578888	0.02595576	0.210164177	Significant Decrease
Isomer 1 of Glutamic Acid	1.066258552	0.359570677	0.484939943	Not Significant
Isomer 1 of Malic Acid	0.839612859	0.376865459	0.491726898	Not Significant
(R)-3-((R)-3-Hydroxybutanoyloxy)butanoic acid	1.30902909	0.108852019	0.301982658	Not Significant
Pyruvic acid	0.812970469	0.010717083	0.166956837	Not Significant
Isomer 1 of Alpha-Ketoglutaric Acid	1.276153846	0.141631238	0.332575052	Not Significant
Isomer 1 of Gamma-Aminobutyric acid	0.941098435	0.755520116	0.641114012	Not Significant
Isomer 2 of Glutamic Acid	0.997620666	0.968273602	0.702378675	Not Significant
Isomer 3 of Alpha-Ketoglutaric Acid	1.186690224	0.162921329	0.346947286	Not Significant
2-Hydroxy-2-Methylbutanedioic Acid	0.911891892	0.211946544	0.390427845	Not Significant
Acetic Acid	0.772026752	0.089981165	0.285432214	Not Significant
Alpha-Ketobutyric Acid	0.925006893	0.377013864	0.491726898	Not Significant
Alpha-Ketoglutaric Acid	1.273113709	0.055304855	0.24299145	Not Significant
Glutamic Acid	0.997539543	0.965330284	0.70199957	Not Significant
Mesaconic Acid	1.280523256	0.139624616	0.331131602	Not Significant
Isomer 1 of Glutamic Acid	1.066258552	0.359570677	0.484939943	Not Significant
4-Hydroxy-4-methylglutamic acid	0.771033654	0.018883169	0.185533142	Not Significant

4-Methyl-L-glutamic acid	0.254573171	0.021142091	0.196902734	Significant Decrease
4-Methylene-L-glutamic acid	1.136290062	0.078428938	0.271830551	Not Significant
L-threo-3-Methylaspartic acid	0.905589343	0.102236778	0.296827663	Not Significant
Pyruvic acid	0.812970469	0.010717083	0.166956837	Not Significant
Isomer 1 of 4-Methylene-L-glutamic acid	1.26344086	0.19701022	0.383323836	Not Significant
Isomer 1 of Acetic Acid	0.913580247	0.545722121	0.570276153	Not Significant
Isomer 1 of Alpha-Ketoglutaric Acid	1.276153846	0.141631238	0.332575052	Not Significant
Isomer 1 of L-threo-3-Methylaspartic acid	0.884754522	0.475421945	0.535096276	Not Significant
Isomer 2 of Glutamic Acid	0.997620666	0.968273602	0.702378675	Not Significant
Isomer 3 of Alpha-Ketoglutaric Acid	1.186690224	0.162921329	0.346947286	Not Significant
Acetic Acid	0.772026752	0.089981165	0.285432214	Not Significant
Glyceric Acid	1.153100775	0.323949982	0.464014017	Not Significant
Malic Acid	0.723878518	0.045969439	0.240081416	Not Significant
Serine	0.802667475	0.094813203	0.290837295	Not Significant
Tyrosine	1.062776025	0.711045669	0.622572083	Not Significant
Isomer 1 of Malic Acid	0.839612859	0.376865459	0.491726898	Not Significant
2-Oxosuberic acid	0.803292894	0.418137309	0.510899753	Not Significant
N-Methyl-L-glutamic acid	0.44364564	0.014109596	0.171457226	Significant Decrease
Pyruvic acid	0.812970469	0.010717083	0.166956837	Not Significant
Isomer 1 of Acetic Acid	0.913580247	0.545722121	0.570276153	Not Significant
Isomer 1 of Glyceric Acid	0.770577933	0.012123515	0.166956837	Not Significant
Isomer 1 of Tyrosine	1.001809955	0.979244114	0.703721121	Not Significant
Isomer 2 of Glyceric Acid	0.735994633	0.001066123	0.136212565	Not Significant
Alanine	1.201553254	0.002911572	0.148733377	Not Significant
Aspartic Acid	0.43049543	0.000376242	0.130023696	Significant Decrease
Malic Acid	0.723878518	0.045969439	0.240081416	Not Significant
Isomer 1 of Malic Acid	0.839612859	0.376865459	0.491726898	Not Significant
Pyruvic acid	0.812970469	0.010717083	0.166956837	Not Significant
Acetylglycine	1.128240581	0.561692585	0.575386502	Not Significant
Tyrosine	1.062776025	0.711045669	0.622572083	Not Significant
N-Acetyl-L-Tyrosine	0.769430725	0.038360449	0.228538487	Not Significant
N-Formyl-4-amino-5-aminomethyl-2-methylpyrimidine	0.576182137	0.002725702	0.148733377	Significant Decrease
Pyridoxal phosphate	0.883443709	0.048904093	0.241458588	Not Significant
Pyruvic acid	0.812970469	0.010717083	0.166956837	Not Significant
Isomer 1 of Acetylglycine	0.776744186	0.310280503	0.458033123	Not Significant
Isomer 1 of Tyrosine	1.001809955	0.979244114	0.703721121	Not Significant
Isomer 2 of Acetylglycine	0.869043959	0.36090121	0.486093193	Not Significant
Pyridoxal	0.899184149	0.302935332	0.454612496	Not Significant
Succinic Semialdehyde	1.412092283	0.176299738	0.359558675	Not Significant
4-Hydroxy-L-threonine / (+)-threo-2-Amino-3,4-dihydroxybutanoic acid	1.66983017	0.116272326	0.307321494	Not Significant
Pyridoxal phosphate	0.883443709	0.048904093	0.241458588	Not Significant
Pyridoxamine phosphate	1.008110848	0.95577732	0.698328883	Not Significant
Aspartic Acid	0.43049543	0.000376242	0.130023696	Significant Decrease
Fumaric Acid	0.533318274	0.006618049	0.156115145	Significant Decrease
Gamma-Aminobutyric acid	1.853543307	0.094477943	0.290837295	Not Significant
Succinic Acid	1.440028902	0.175249313	0.358765104	Not Significant
Succinic Semialdehyde	1.412092283	0.176299738	0.359558675	Not Significant
Isomer 1 of Fumaric Acid	0.540578888	0.02595576	0.210164177	Significant Decrease
2,3,6-Trihydroxypyridine	1.13723326	0.369601224	0.489962197	Not Significant
6-Oxo-1,4,5,6-tetrahydronicotinic acid	1.221146953	0.14143622	0.332575052	Not Significant

Iminoaspartic acid	0.926695842	0.711396959	0.622572083	Not Significant
Maleamic acid	0.786750616	0.081423486	0.274361746	Not Significant
Pyruvic acid	0.812970469	0.010717083	0.166956837	Not Significant
Isomer 1 of Gamma-Aminobutyric acid	0.941098435	0.755520116	0.641114012	Not Significant
Aspartic Acid	0.43049543	0.000376242	0.130023696	Significant Decrease
Uracil	0.787630167	0.075827494	0.270139896	Not Significant
Valine	1.079760718	0.232964721	0.403460817	Not Significant
2,3-Dihydroxy-3-methylbutanoic acid / (R)-2,3-Dihydroxy-3-methylbutanoic acid	1.00215783	0.975197822	0.702864616	Not Significant
Pyruvic acid	0.812970469	0.010717083	0.166956837	Not Significant
Lysine	1.251472116	0.043063596	0.236691698	Not Significant
N6-Acetyl-Lysine	1.256925996	0.037968822	0.228538487	Not Significant
Pimelate / 3-Methyladipic Acid	1.332567301	0.278045381	0.43753334	Not Significant
4-Hydroxybenzoic acid	0.831353231	0.224416148	0.398187279	Not Significant
4-Aminobenzoic acid	0.909681611	0.158744435	0.344287374	Not Significant
7-Cyano-7-carbaguanine	1.662354463	0.025150358	0.209908759	Significant Increase
Cyanamide	1.111689815	0.053352546	0.241458588	Not Significant
5-Aminolevulinic acid	0.846774194	0.081182627	0.274139597	Not Significant
Acetylglycine	1.128240581	0.561692585	0.575386502	Not Significant
Glutamic Acid	0.997539543	0.965330284	0.70199957	Not Significant
Threonine	0.602526203	0.068006986	0.258168326	Not Significant
Isomer 1 of Glutamic Acid	1.066258552	0.359570677	0.484939943	Not Significant
(3Z)-Phycocyanobilin	0.75336893	0.056906576	0.244746612	Not Significant
Isomer 1 of Acetylglycine	0.776744186	0.310280503	0.458033123	Not Significant
Isomer 2 of Acetylglycine	0.869043959	0.36090121	0.486093193	Not Significant
Isomer 2 of Glutamic Acid	0.997620666	0.968273602	0.702378675	Not Significant
Pyruvic acid	0.812970469	0.010717083	0.166956837	Not Significant
Tryptophan	1.031818182	0.852319068	0.671511874	Not Significant
Gibberellin A34-catabolite	0.946063506	0.777211947	0.649198882	Not Significant
Gibberellin A51-catabolite	1.155923133	0.472255743	0.532933308	Not Significant
Isomer 1 of Gibberellin A51-catabolite	1.021167883	0.881475468	0.679487121	Not Significant
Dihydrophaseic acid / Epidihydrophaseic acid	1.155343241	0.638506403	0.59365732	Not Significant
5'-Methylthioadenosine	0.747024718	0.257828859	0.420627925	Not Significant
Acetic Acid	0.772026752	0.089981165	0.285432214	Not Significant
Adenine	0.846973973	0.35943494	0.484939943	Not Significant
Isomer 1 of Acetic Acid	0.913580247	0.545722121	0.570276153	Not Significant
Isomer 1 of Adenine	0.868716931	0.304930735	0.455716044	Not Significant
Glutamic Acid	0.997539543	0.965330284	0.70199957	Not Significant
Isomer 1 of Glutamic Acid	1.066258552	0.359570677	0.484939943	Not Significant
L-Glutamine / D-Glutamine	1.008454515	0.926636664	0.690759726	Not Significant
Isomer 2 of Glutamic Acid	0.997620666	0.968273602	0.702378675	Not Significant
Acetic Acid	0.772026752	0.089981165	0.285432214	Not Significant
Homoserine	0.722604705	0.01955649	0.189453493	Not Significant
Serine	0.802667475	0.094813203	0.290837295	Not Significant
Succinic Acid	1.440028902	0.175249313	0.358765104	Not Significant
Isomer 1 of Homoserine	0.932373619	0.694047788	0.616741892	Not Significant
L-Homocysteine	0.923754659	0.677968182	0.609188801	Not Significant
S-Sulfanylglutathione	1.077805332	0.696357432	0.61705703	Not Significant
Isomer 1 of Acetic Acid	0.913580247	0.545722121	0.570276153	Not Significant
Adipic Acid	1.369964664	0.113051575	0.306811535	Not Significant
E-Aminocaproic Acid	0.816874764	0.465859782	0.530591322	Not Significant

Phenylalanine	0.914249283	0.436039882	0.518363083	Not Significant
Tyrosine	1.062776025	0.711045669	0.622572083	Not Significant
3,4-Dihydroxystyrene	0.903822816	0.233523865	0.403460817	Not Significant
4-Hydroxystyrene	1.039151116	0.367033736	0.48829422	Not Significant
5-Hydroxyconiferaldehyde	1.505249344	0.082173733	0.275326931	Not Significant
Ferulic acid	0.848677923	0.311571681	0.458069476	Not Significant
Spermidine	0.342320966	0.065268951	0.252446023	Not Significant
Isomer 1 of 3,4-Dihydroxystyrene	0.879259014	0.104232107	0.297610096	Not Significant
Isomer 1 of Tyrosine	1.001809955	0.979244114	0.703721121	Not Significant
Isomer 2 of 3,4-Dihydroxystyrene	0.964565786	0.795628492	0.654651054	Not Significant
Resveratrol	0.93575346	0.71283019	0.623284193	Not Significant
Tyrosine	1.062776025	0.711045669	0.622572083	Not Significant
3-(4-Hydroxyphenyl)pyruvic acid	0.84588934	0.070529503	0.262519762	Not Significant
Isomer 1 of Tyrosine	1.001809955	0.979244114	0.703721121	Not Significant
Alloisoleucine	0.938195303	0.653398811	0.598764957	Not Significant
Lysine	1.251472116	0.043063596	0.236691698	Not Significant
Phenylalanine	0.914249283	0.436039882	0.518363083	Not Significant
Phenylpyruvic Acid	1.049965302	0.769906342	0.646804786	Not Significant
Pipecollinic Acid	1.205513784	0.056957161	0.244746612	Not Significant
Isomer 1 of Pipecollinic Acid	1.092413236	0.501769374	0.548810252	Not Significant
(S)-2,3,4,5-Tetrahydropyridine-2-carboxylic acid	1.088671612	0.33211234	0.468490149	Not Significant
5-Aminopentanal	1.113561191	0.157384229	0.343585288	Not Significant
6-Amino-2-oxohexanoic acid	1.037185563	0.660588046	0.601795155	Not Significant
Delta1-Piperideine-2-carboxylic acid	1.044580153	0.813691685	0.660112518	Not Significant
L-2-Aminoadipate 6-semialdehyde / Allysine	1.155949367	0.04554703	0.240081416	Not Significant
Isomer 1 of (S)-2,3,4,5-Tetrahydropyridine-2-carboxylic acid	1.148992402	0.166883176	0.350567756	Not Significant
Isomer 1 of 5-Aminopentanal	1.026910657	0.147138114	0.335741017	Not Significant
Isomer 1 of Delta1-Piperideine-2-carboxylic acid	1.10833079	0.002716502	0.148733377	Not Significant
Isomer 2 of (S)-2,3,4,5-Tetrahydropyridine-2-carboxylic acid	1.202155333	0.03034741	0.215914363	Not Significant
Tyrosine	1.062776025	0.711045669	0.622572083	Not Significant
2-Descarboxy-cyclo-dopa	0.881626351	0.424019487	0.512032436	Not Significant
Betalamic acid	1.214496418	0.334261132	0.469784104	Not Significant
Isomer 1 of Tyrosine	1.001809955	0.979244114	0.703721121	Not Significant
Alloisoleucine	0.938195303	0.653398811	0.598764957	Not Significant
Leucine	0.977616646	0.822884278	0.663572912	Not Significant
Methionine	0.870646766	0.145357826	0.334756842	Not Significant
Phenylalanine	0.914249283	0.436039882	0.518363083	Not Significant
Tryptophan	1.031818182	0.852319068	0.671511874	Not Significant
Tyrosine	1.062776025	0.711045669	0.622572083	Not Significant
Valine	1.079760718	0.232964721	0.403460817	Not Significant
Isomer 1 of Methionine	1.243744956	0.170796169	0.354235151	Not Significant
Isomer 1 of Tyrosine	1.001809955	0.979244114	0.703721121	Not Significant
4-Chloro-L-lysine	1.242139649	0.078668012	0.271830551	Not Significant
L-Propargylglycine	1.154605263	0.19731462	0.383323836	Not Significant
Isomer 1 of L-Propargylglycine	1.176300578	0.025881486	0.210164177	Not Significant
3-[(1R,2S,5R,6S)-5-Hydroxy-7-oxabicyclo[4.1.0]heptan-2-yl]-2-oxopropanoic acid	1.288743254	0.172657524	0.35678616	Not Significant

3-[(1R,2S,5R,6S)-5-Hydroxy-7-oxabicyclo[4.1.0]heptan-2-yl]-2-oxopropanoic acid	1.288743254	0.172657524	0.35678616	Not Significant
Pentalenic acid	1.359970403	0.061173539	0.249978299	Not Significant
Oleic Acid / Vaccenic Acid	1.126636388	0.717960741	0.624438801	Not Significant
Citric Acid / Isocitric Acid	0.496081277	0.053115803	0.241458588	Not Significant
Protocatechuic acid	1.351843625	0.353682316	0.482092101	Not Significant
Salicylic Acid	1.171747967	0.244864356	0.412198411	Not Significant
Spermidine	0.342320966	0.065268951	0.252446023	Not Significant
2,2-Dimethylsuccinic Acid	1.06773743	0.164801838	0.348790348	Not Significant
2-Furoic Acid	0.910232843	0.283766002	0.44204754	Not Significant
2-Hydroxycaproic Acid / Hydroxyisocaproic Acid / 2-Ethyl-2-Hydroxybutyric Acid	0.993087558	0.924091196	0.690405441	Not Significant
3-Hydroxymethylglutaric Acid	1.060818713	0.246411438	0.412268944	Not Significant
5-Hydroxymethyluracil	0.806214501	0.174856931	0.358765104	Not Significant
Azelaic Acid	1.156010929	0.478655159	0.535679059	Not Significant
Dimethylmalonic Acid / Ethylmalonic Acid	1.215153268	0.15822231	0.344030474	Not Significant
Guanidine	0.305743243	0.404096108	0.505476935	Not Significant
Hydroxyoctanoic Acid	1.088617074	0.136370889	0.330728592	Not Significant
Indole-3-carboxylic acid	1.333738356	0.070344818	0.262282226	Not Significant
Methylsuccinic Acid	1.12863315	0.256645939	0.420318255	Not Significant
N-Methyl-A-Aminoisobutyric Acid	1.026782346	0.908619913	0.688395849	Not Significant
Phthalic Acid	1.452106174	0.223883126	0.398187279	Not Significant
Sebacic Acid	1.169000637	0.211203939	0.390427845	Not Significant
Undecanedioic Acid	0.95278865	0.827584726	0.664782648	Not Significant
(-)-trans-3,4-Dihydro-4,8-dihydroxy-3-methyl-1H-2-benzopyran-1-one	1.845768653	0.029395187	0.213612074	Significant Increase
(6R)-6-(L-Erythro-1,2-Dihydroxypropyl)-5,6,7,8-tetrahydro-4a-hydroxypterin	1.155284553	0.052853838	0.241458588	Not Significant
(S)-9-Hydroxy-10-undecenoic acid	1.143858355	0.356387859	0.484412555	Not Significant
1H-Indole-3-methanamine	0.957910015	0.778745254	0.649198882	Not Significant
2(N)-Methyl-norsalsolinol	1.171509568	0.205188535	0.385774501	Not Significant
2,2,6,6-Tetramethyl-4-piperidinone	1.392943063	0.012108517	0.166956837	Not Significant
2,4,6-Trimethylphenol	0.971340524	0.875147618	0.677988408	Not Significant
2,4-Diamino-6-hydroxypyrimidine	0.683844947	0.159803665	0.34436341	Not Significant
2,6-Dimethyl-1,4-benzenediol	0.651140317	0.043840778	0.236887617	Significant Decrease
2,8-Dihydroxyadenine	1.506014435	0.052242758	0.241458588	Not Significant
2-Amino-2-methyl-1,3-propanediol	0.841216216	0.494573305	0.5440351	Not Significant
2-Aminoacetophenone	1.283743475	0.250885078	0.417114804	Not Significant
2-Hydroxy-4-imino-2,5-cyclohexadienone	1.085311318	0.027269764	0.211124116	Not Significant
2-Hydroxy-4-methylbenzaldehyde	0.957151665	0.606794163	0.587273457	Not Significant
2-Hydroxydecanedioic acid	1.294050777	0.027365929	0.211124116	Not Significant
2-Hydroxydecanoic acid	1.050460077	0.843724416	0.669182012	Not Significant
2-Methyl-3-oxopropanoic acid	1.307606264	0.136558902	0.330728592	Not Significant
2-Octenedioic acid	1.049201278	0.601100333	0.586520137	Not Significant
2-Pyrrolidineacetic acid	0.819761129	0.214486808	0.39178146	Not Significant
3,4-Dihydroxyphenylvaleric acid	0.769324407	0.104139151	0.297610096	Not Significant
3-Deoxy-D-glycero-D-galacto-2-nonulosonic acid	1.42154767	0.150067284	0.339313157	Not Significant
3-Hydroxy-4-aminopyridine	0.875468867	0.394025534	0.50237098	Not Significant
3-Hydroxydodecanoic acid / (R)-3-Hydroxydodecanoic acid	1.096276417	0.521286448	0.560550839	Not Significant
3-Hydroxynonanoic acid	0.935170686	0.195766822	0.381683741	Not Significant

3-Methyl-1-butylamine	0.766988008	0.22584913	0.399423482	Not Significant
3-Oxododecanoic acid	1.090342679	0.098992163	0.292664763	Not Significant
3-Oxoglutaric acid	1.172695449	0.150034272	0.339313157	Not Significant
4-(3-Pyridyl)-3-butenoic acid	1.022390755	0.915443979	0.688911723	Not Significant
4-Amino-2-methylenebutanoic acid	1.016634677	0.838701139	0.667393279	Not Significant
4-Aminobiphenyl	1.045897644	0.758878122	0.641263834	Not Significant
4-Carboxypyrazole	1.368588469	0.151495334	0.340130673	Not Significant
4-Hydroxybenzyl alcohol	0.902969247	0.116114678	0.307279086	Not Significant
4-Hydroxybenzylamine	0.933353169	0.596352791	0.584501155	Not Significant
4-Hydroxypiperidine	1.0914037	0.183274864	0.368246717	Not Significant
5-Heptyltetrahydro-2-oxo-3-furancarboxylic acid	1.43460076	0.162308081	0.346920677	Not Significant
6-Hydroxydopamine	0.898797875	0.656576492	0.600128052	Not Significant
8-Hydroxyadenine	1.083023544	0.804539843	0.656828991	Not Significant
A-Ketoglutaric acid oxime	1.294250792	0.089994595	0.285432214	Not Significant
Acetylhydrazine	0.831829897	0.104673032	0.297648105	Not Significant
Adenosine 2'-phosphate	0.783946701	0.145542363	0.334756842	Not Significant
Avenanthramide 1p	0.913473956	0.498890154	0.546763452	Not Significant
Dodecanedioic acid	1.307747489	0.09023341	0.285432214	Not Significant
FAPy-adenine	0.949775785	0.79583847	0.654651054	Not Significant
Glycidic acid	0.754716981	0.007507051	0.156115145	Not Significant
Hexylamine	1.101119538	0.114940277	0.307279086	Not Significant
Iminodiacetic acid	1.001984127	0.976363307	0.702864616	Not Significant
Indoleacrylic acid	0.96994648	0.83418728	0.666489837	Not Significant
Mesalazine	1.034046693	0.931382114	0.692248214	Not Significant
Monoisobutyl phthalic acid	1.239292731	0.174510782	0.358765104	Not Significant
Morpholine	0.985229415	0.949466445	0.695826472	Not Significant
N-Carboxyethyl-g-aminobutyric acid	0.818307906	0.398546732	0.502817679	Not Significant
Norfuraneol	0.835820896	0.224785436	0.398516664	Not Significant
Octadecanedioic acid	1.37394636	0.481256732	0.537758553	Not Significant
Pyrazin-2-carboxylic acid	1.104399702	0.207780331	0.387565583	Not Significant
Quinaldic acid	0.967103259	0.871068448	0.677452733	Not Significant
Salsolinol 1-carboxylic acid	0.774334252	0.345871758	0.475773882	Not Significant
Umbelliferone	0.957833515	0.832546127	0.665423608	Not Significant
alpha-CEHC	1.082831325	0.524546674	0.562198491	Not Significant
alpha-Cyano-4-hydroxycinnamic acid	1.098631099	0.400468726	0.504097006	Not Significant
m-Tyramine	0.552727273	0.008466047	0.162966298	Significant Decrease
trans-2-Hexyl-1-cyclopropaneacetic acid	1.035043353	0.855501681	0.672135644	Not Significant
Isomer 1 of (6R)-6-(L-Erythro-1,2-Dihydroxypropyl)-5,6,7,8-tetrahydro-4a-hydroxypterin	0.909722222	0.576382868	0.580636442	Not Significant
Isomer 1 of 2,2-Dimethylsuccinic Acid	1.084461867	0.540793451	0.56884236	Not Significant
Isomer 1 of 3-Hydroxynonanoic acid	0.903414065	0.754638714	0.641114012	Not Significant
Isomer 1 of Dodecanedioic acid	1.209404849	0.431916107	0.515543428	Not Significant
Isomer 1 of N-Carboxyethyl-g-aminobutyric acid	0.579078801	0.365937699	0.488177413	Not Significant
Isomer 1 of Norfuraneol	1.038782051	0.572378225	0.578509897	Not Significant
Isomer 2 of 2-Oxoglutaric acid	1.274971076	0.182740957	0.36819673	Not Significant
Isomer 2 of Dodecanedioic acid	1.123055163	0.53070044	0.56463311	Not Significant
