# Supplementary material for: Evaluating the effects of synthetic POM cycles and NAD+ kinase expression on fatty alcohol production in Saccharomyces cerevisiae
Source: PLoS One. 2025 Sep 29;20(9):e0333299. doi: 10.1371/journal.pone.0333299 (PMC12478946; doi:10.1371/journal.pone.0333299)
Supplement: S3 Table — A pathway impact table based on metabolite differences observed between S. cerevisiae strains harboring an empty vector of the NC1 POM cycle. (DOCX) [file pone.0333299.s006.docx]

**Table S3, Pathway Impact table**

Name Total Cmpd Hits Raw p -log10(p) Holm Adjust FDR Impact

Monobactam biosynthesis 4 1 7.57E-05 4.12E+00 3.56E-03 3.56E-03 0.00

Lipoic acid metabolism 25 2 2.96E-04 3.53E+00 1.36E-02 6.96E-03 0.00

**Arginine biosynthesis** 18 9 4.46E-04 3.35E+00 2.01E-02 6.99E-03 **0.47**

Lysine biosynthesis 16 7 7.73E-04 3.11E+00 3.40E-02 8.65E-03 0.14

**Alanine, aspartate and glutamate**

**metabolism**  22 11 9.21E-04 3.04E+00 3.96E-02 8.65E-03 **0.92**

**Glutathione metabolism** 26 6 1.49E-03 2.83E+00 6.27E-02 1.17E-02 **0.48**

beta-Alanine metabolism 11 3 1.90E-03 2.72E+00 7.80E-02 1.28E-02 0.00

**Pyruvate metabolism** 24 5 3.55E-03 2.45E+00 1.42E-01 2.08E-02 **0.41**

**Citrate cycle (TCA cycle)** 20 7 4.81E-03 2.32E+00 1.88E-01 2.51E-02 **0.35**

**Glycine, serine, threonine metabolism** 32 14 5.99E-03 2.22E+00 2.28E-01 2.81E-02 **0.44**

Tyrosine metabolism 9 7 6.67E-03 2.18E+00 2.47E-01 2.85E-02 0.00

Glycolysis / Gluconeogenesis 24 2 1.18E-02 1.93E+00 4.25E-01 4.62E-02 0.10

**Tryptophan metabolism** 30 7 1.57E-02 1.80E+00 5.49E-01 5.67E-02 **0.53**

Sulfur metabolism 13 4 1.76E-02 1.75E+00 6.00E-01 5.92E-02 0.15

Lysine degradation 17 4 2.12E-02 1.67E+00 6.98E-01 6.19E-02 0.33

C5-Branched dibasic acid metabolism 4 2 2.13E-02 1.67E+00 6.98E-01 6.19E-02 0.0

Cyanoamino acid metabolism 12 6 2.24E-02 1.65E+00 6.98E-01 6.19E-02 0.00

Thiamine metabolism 18 1 2.46E-02 1.61E+00 7.39E-01 6.22E-02 0.00

Taurine, hypotaurine metabolism 7 4 2.51E-02 1.60E+00 7.39E-01 6.22E-02 0.00

**Cysteine, methionine metabolism** 41 20 2.84E-02 1.55E+00 7.97E-01 6.69E-02 **0.64**

**Glyoxylate and dicarboxylate**

**metabolism** 26 10 3.38E-02 1.47E+00 9.12E-01 7.56E-02 0.34

D-Amino acid metabolism 4 3 3.56E-02 1.45E+00 9.25E-01 7.60E-02 0.00

**Butanoate metabolism** 14 5 4.02E-02 1.40E+00 1.00E+00 8.22E-02 **0.80**

Arginine and proline metabolism 25 10 4.52E-02 1.34E+00 1.00E+00 8.51E-02 0.54

Valine, leucine, isoleucine biosynthe- 20 8 4.53E-02 1.34E+00 1.00E+00 8.51E-02 0.33

sis

Methane metabolism 23 2 4.84E-02 1.32E+00 1.00E+00 8.75E-02 0.26

Atrazine degradation 4 1 5.11E-02 1.29E+00 1.00E+00 8.89E-02 0.00

Sphingolipid metabolism 13 1 6.18E-02 1.21E+00 1.00E+00 1.04E-01 0.00

Propanoate metabolism 18 2 6.97E-02 1.16E+00 1.00E+00 1.13E-01 0.00

Pantothenate and CoA biosynthesis 22 3 8.60E-02 1.07E+00 1.00E+00 1.35E-01 0.04

Biosynthesis of plant secondary

metabolites 4 1 1.20E-01 9.21E-01 1.00E+00 1.82E-01 0.00

Folate biosynthesis 23 1 1.29E-01 8.88E-01 1.00E+00 1.90E-01 0.06

Vitamin B6 metabolism 12 5 1.42E-01 8.49E-01 1.00E+00 1.97E-01 0.86

Nicotinate, nicotinamide metabolism 12 2 1.43E-01 8.46E-01 1.00E+00 1.97E-01 0.00

Glycerophospholipid metabolism 32 2 1.58E-01 8.02E-01 1.00E+00 2 .12E-01 0.02

Phenylalanine, tyrosine, tryptophan

biosynthesis 21 6 1.80E-01 7.44E-01 1.00E+00 2.35E-01 0.1

Ubiquinone and other terpenoid-quinone

biosynthesis 16 3 1.90E-01 7.21E-01 1.00E+00 2.42E-01 0.0

Pyrimidine metabolism 35 5 2.13E-01 6.72E-01 1.00E+00 2.63E-01 0.10

Porphyrin metabolism 23 2 2.35E-01 6.30E-01 1.00E+00 2.83E-01 0.03

Purine metabolism 65 6 2.52E-01 5.99E-01 1.00E+00 2.96E-01 0.05

Glycerolipid metabolism 14 1 3.23E-01 4.90E-01 1.00E+00 3.71E-01 0.07

Phenylalanine metabolism 5 2 5.87E-01 2.32E-01 1.00E+00 6.57E-01 1.00

Valine, leucine, isoleucine degrada- 18 3 6.02E-01 2.21E-01 1.00E+00 6.58E-01 0.00

tion

Biosynthesis of unsaturated fatty acids 23 1 7.04E-01 1.52E-01 1.00E+00 7.52E-01 0.00

Histidine metabolism 16 2 7.58E-01 1.21E-01 1.00E+00 7.91E-01 0.00

Carbapenem biosynthesis 3 1 9.65E-01 1.55E-02 1.00E+00 9.86E-01 0.00

Nitrogen metabolism 6 2 9.95E-01 2.16E-03 1.00E+00 9.95E-01 0.00
